# The Crosstalk Mechanisms Between Ferroptosis and Pyroptosis and Their Applications in Diseases: From Molecular Networks to Clinical Strategies

**DOI:** 10.1111/jcmm.71138

**Published:** 2026-04-21

**Authors:** Wei‐Yi Zhao, Lu‐yao Li, Fang‐wang Ye, Jin‐Wei Zhao

**Affiliations:** ^1^ Department of Clinical Pathalogy of Yanbian Hospital of Yanbian University Yanbian University Yanbian China; ^2^ Department of Hepatopancreatobiliary Surgery of Second Hospital of Jilin University Jilin University Changchun China

**Keywords:** crosstalk, ferroptosis, multi‐target drugs, nanocarrier‐based drug delivery systems, precision medicine, pyroptosis

## Abstract

Ferroptosis and pyroptosis are two distinct forms of regulated cell death that play crucial roles in cancer, neurodegeneration, and inflammatory diseases. Ferroptosis is characterised by iron‐dependent lipid peroxidation, while pyroptosis is an inflammatory cell death mediated by gasdermin proteins. Recent studies reveal extensive crosstalk between these pathways. This review establishes the first hierarchical framework coupling the autophagy bridge function (ferritinophagy‐mitophagy‐cyclic GMP‐AMP synthase (cGAS)‐stimulator of interferon genes (STING) axis) with the p53/signal transducer and activator of transcription 3 (STAT3)/Nuclear factor erythroid 2–related factor 2 (NRF2) transcriptional hub, creating a unified decision‐making network absent in prior reviews. Crosstalk mechanisms include the reactive oxygen species (ROS)‐NOD‐like receptor protein 3 (NLRP3) positive feedback loop, caspase cross‐activation, and iron metabolism‐inflammasome integration. Preclinically, the transferrin‐targeted nanosystem Tf‐LipoMof@PL increased intratumoral iron/ROS 3–5‐fold, inducing robust antitumour immunity, while Ginsenoside Rh3 suppressed colorectal cancer growth in vivo via STAT3/p53/NRF2‐mediated dual death induction. We critically address STAT3's paradoxical roles—promoting Gasdermin E (GSDME)‐mediated pyroptosis in oesophageal cancer while suppressing NLRP3 via suppressor of cytokine signalling 3 (SOCS3) feedback in acute respiratory distress syndrome (ARDS)—highlighting cell type‐specific feedback architectures that dictate phenotypic outcomes. For therapeutic translation, we propose a Translational Priority Matrix ranking nanodelivery systems (Tf‐LipoMof@PL) and dual‐function small molecules (N6F11) as the highest priority for intrahepatic cholangiocarcinoma (iCCA)/triple‐negative breast cancer (TNBC), while deprioritising metal photosensitizers pending resolution of cardiac retention toxicity (0.8 μg/g myocardium in Good Laboratory Practice (GLP) studies). The “registration gap” stems from iron burst‐release (> 80% within 30 min) and species‐specific biomarker failures. We advocate replacing murine malondialdehyde (MDA)/glutathione (GSH) ratios with human‐anchored metrics (ferritin heavy chain 1 (FTH1)/solute carrier family 40 member 1 (SLC40A1) expression, serum ferritin) and propose a “Cross‐Death AI Platform” integrating network pharmacology (OmniPath/STRING), GraphSAGE deep learning (AlphaFold2 structures), and organoid validation to stratify patients and predict optimal drug combinations. By resolving spatiotemporal heterogeneity and implementing AI‐guided precision medicine, we can transform multi‐target interventions from empirical strategies into rational, patient‐specific regimens, bridging the gap between preclinical promise and clinical success in cancers and neurodegenerative diseases.

**Abbreviations**
4‐HNE: 4‐hydroxynonenal
ADP‐ribose: DNA repair enzyme poly
AML: acute myeloid leukaemia
ARDS: acute respiratory distress syndrome
ASC: apoptosis‐associated speck‐like protein containing a caspase recruitment domain
ATM: ataxia telangiectasia mutated
BSA: bovine serum albumin
Ca^2+^
: calcium ion
cGAS: cyclic GMP‐AMP synthase
CO: carbon monoxide
CRC: colorectal cancer
CRC: colorectal cancer
DAMPs: damage‐associated molecular patterns
DAMPs: damage‐associated molecular patterns
DUBs: deubiquitinases
DUBs: deubiquitinating enzyme
ESCC: oesophageal squamous cell carcinoma
ETC: electron transport chain
FA: folic acid
Fe^2+^
: ferrous ions
FGL1: fibrinogen‐like protein 1
FTH1: ferritin heavy chain 1
GLP: Good Laboratory Practice
GPX4: glutathione peroxidase 4
GRh3: Ginsenoside Rh3
GSDMD: Gasdermin D
GSDME: Gasdermin E
GSH: glutathione
H_2_O_2_
: hydrogen peroxide
HCC: hepatocellular carcinoma
HO‐1: heme oxygenase‐1
HO‐1: heme oxygenase‐1
ICB: immune checkpoint blockade
iCCA: intrahepatic cholangiocarcinoma
ICD: immunogenic cell death
IL‐1β: interleukin‐1β
IrC/IrF: iridium(III) photosensitizer
IRF3: interferon regulatory factor 3
K^+^
: intracellular potassium ions
LIP: labile iron ions
LPS: lipopolysaccharide
MDA: malondialdehyde
MOF: metal–organic framework
MOFs: metal–organic frameworks
MPs: microplastics
MPT: mitochondrial permeability transition
mtDNA: mitochondrial DNA
MUFAs: monounsaturated fatty acids
MVA: metabolites in the mevalonate
NAC: N‐acetylcysteine
NLRP3: NOD‐like receptor protein 3
NOX: NADPH oxidase
NOX4: NADPH oxidase 4
Nrf2: nuclear factor erythroid 2‐related factor 2
OH: hydroxyl radicals
OXPHOS: oxidative phosphorylation
PAMP: pathogen‐associated molecular pattern
PARP1: polymerase 1
PL: H_2_O_2_ donor piperine
PLA_2_
: phospholipase A_2_
PRRs: pattern recognition receptors
PUFAs: polyunsaturated fatty acids
ROS: reactive oxygen species
SLC7A11: solute carrier family 7 member 11
SOCS3: suppressor of cytokine signalling 3
STAT3: signal transducer and activator of transcription 3
STING: stimulator of interferon genes
System Xc^−^
: glutamate antiporter
TBK1: TANK‐binding kinase 1
TCA: tricarboxylic acid cycle
TLR4: Toll‐like receptor 4 pathway
TNBC: triple‐negative breast cancer
TPL: triptolide
VDAC: voltage‐dependent anion channel

## 1 Introduction

Cell death is a fundamental biological process that maintains homeostasis in the body, and its dysregulation is closely associated with the development of various diseases, including cancer, neurodegenerative diseases, and inflammatory conditions [1, 2]. Historically, apoptosis and necrosis have been the primary focuses of research into disease mechanisms. However, with the discovery of new types of programmed cell death, ferroptosis and pyroptosis have gradually emerged as cutting‐edge research topics in the field of life sciences [3, 4].

Ferroptosis is characterised by iron‐dependent lipid peroxidation and is regulated by key molecules such as glutathione peroxidase 4 (GPX4) and solute carrier family 7 member 11 (SLC7A11) [4]. Pyroptosis, on the other hand, is an inflammatory form of cell death mediated by caspase‐1, caspase‐4, caspase‐5 and caspase‐11 (non‐canonical pathway) or Gasdermin E (GSDME) by Caspase‐3 (often downstream of apoptosis signalling), a family of proteins. It relies on the cleavage of Gasdermin D (GSDMD), leading to the release of pro‐inflammatory factors such as interleukin‐1β (IL‐1β) [2]. Although there are significant differences in the regulatory mechanisms and pathological effects of these two processes, recent studies have revealed that they engage in deep crosstalk within the networks of metabolic reprogramming, oxidative stress, and inflammatory signalling [1, 5]. This crosstalk provides a new perspective for understanding disease mechanisms and developing novel therapeutic strategies.

Several key intersections between ferroptosis and pyroptosis have been identified. For example, BRCA1/BRCA2‐containing complex subunit 3 (BRCC36)‐mediated deubiquitination of 3‐hydroxy‐3‐methylglutaryl‐CoA reductase (HMGCR) can simultaneously inhibit ferroptosis and activate pyroptosis [6]. Additionally, iron overload can amplify ROS signalling through the Fenton reaction, leading to the oxidation of mitochondrial Tom20 and activation of the pyroptosis pathway [7]. These findings not only reveal the complexity of the cell death network but also suggest that targeting these cross‐mechanisms may overcome the limitations of single‐pathway targeted therapies, such as tumour resistance and the amplification of neuroinflammatory cascades.

However, the translational application in this field still faces multiple challenges. The interaction between ferroptosis and pyroptosis is dependent on time, space and cell type, necessitating the development of dynamic regulatory strategies [6]. Existing drugs are mostly single‐targeted and cannot achieve precise intervention in multiple pathways. Moreover, nanodelivery systems need to be optimised to enhance therapeutic selectivity and safety [7].

This review aims to systematically summarise the core molecular network of the cross‐mechanisms between ferroptosis and pyroptosis, evaluate the latest research progress of small‐molecule drugs, metal complexes, and nanodelivery systems targeting this network, and explore their potential applications in cancer. This article is intended to provide a theoretical basis for multi‐target drug design and translational research, and to promote the in‐depth study of cross‐death mechanisms in the era of precision medicine.

## 2 Overview of the Molecular Mechanisms of Ferroptosis and Pyroptosis

### 2.1 Ferroptosis

#### 2.1.1 Core Pathway

Ferroptosis is an iron‐dependent form of programmed cell death. During ferroptosis, iron ions within the cell participate in the Fenton reaction, catalysing the conversion of hydrogen peroxide to hydroxyl radicals (·OH), which are highly reactive. These radicals can attack polyunsaturated fatty acids (PUFAs) in the cell membrane, triggering lipid peroxidation. Lipid peroxidation products, such as MDA and 4‐hydroxynonenal (4‐HNE), accumulate and damage the integrity of the cell membrane. As lipid peroxidation intensifies, the fluidity of the cell membrane decreases while its permeability increases, eventually leading to membrane rupture and the release of cellular contents, thus inducing ferroptosis [8].

#### 2.1.2 Key Regulatory Factors

The GSH‐GPX4 antioxidant axis represents the central negative regulatory mechanism of ferroptosis, where GPX4 utilises GSH to detoxify lipid peroxides; interruption of this pathway—whether through direct GPX4 inhibition or cysteine deprivation via system xc^−^ blockade—eliminates cellular protection against lipid peroxidation and induces ferroptotic cell death [9]. Therefore, any factors that lead to decreased GSH levels or inhibited GPX4 activity may trigger ferroptosis.

#### 2.1.3 Triggering Mechanisms

Ferroptosis can be triggered through various mechanisms. One common mechanism is the inhibition of the glutamate/cystine antiporter xc^−^, which reduces cellular cystine uptake. Cystine is a key precursor for GSH synthesis, and its reduced uptake leads to decreased GSH levels and subsequent inhibition of GPX4 activity. Additionally, certain small‐molecule compounds can directly target GPX4 to inhibit its activity, thereby inducing ferroptosis. These findings provide an important theoretical basis for the development of therapeutic strategies targeting ferroptosis [10].

As demonstrated by Zhao et al., the hepatoprotective effects exerted by functional foods and bioactive constituents are also predominantly mediated through the selective suppression of ferroptosis via the GSH‐GPX4 antioxidant axis and its associated regulatory networks. This mechanistic appreciation underscores the necessity of sustained in‐depth investigation into ferroptosis‐related pathways [11].

### 2.2 Molecular Mechanism of Pyroptosis

#### 2.2.1 Classical Pathway

Pyroptosis is primarily mediated by the activation of inflammasome complexes. Inflammasome complexes are innate immune signalling platforms assembled by pattern recognition receptors (PRRs) upon detection of pathogen‐derived or endogenous danger signals. For instance, the NLRP3 inflammasome plays a crucial role in this process. NLRP3, apoptosis‐associated speck‐like protein containing a caspase recruitment domain (ASC), and pro‐Caspase‐1 are activated and assembled into the canonical NLRP3 inflammasome. Activated Caspase‐1 promotes the conversion of pro‐IL‐1β and pro‐IL‐18 to IL‐1β and IL‐18, and it cleaves Gasdermin D (GSDMD) to release the N‐terminal fragment of Gasdermin D (NT‐GSDMD), which forms pores in the cell membrane, leading to cytokine release and cell death [12].

#### 2.2.2 Non‐Canonical Pathway

When the canonical NLRP3 pathway is blocked, ATP can induce pyroptosis in macrophages via the Caspase‐3/GSDME axis. Additionally, in the non‐canonical inflammasome pathway, lipopolysaccharide (LPS) from Gram‐negative bacteria directly binds to Caspase‐11 (in mice) or Caspase‐4 and Caspase‐5 (in humans), inducing the oligomerisation and activation of Caspase [13, 14].

#### 2.2.3 The Role of Gasdermin Family Proteins

Upon activation of Caspase‐1, Caspase‐11, Caspase‐4 or Caspase‐5, Gasdermin D is cleaved, generating an N‐terminal fragment that induces pyroptosis, while the C‐terminal fragment acts as a negative regulator, auto‐inhibiting Gasdermin D. The N‐terminal fragment of Gasdermin D oligomerizes in the membrane to form pores, leading to the leakage of cellular contents, activation of the inflammatory response, and cell swelling and rupture, which are typical features of pyroptosis [2, 15]. Similar to Gasdermin D, GSDME can also be cleaved by Caspase, producing an N‐terminal fragment with pore‐forming ability that induces pyroptosis [15]. The activation of Gasdermin E is often associated with the apoptosis pathway, especially after Caspase‐3 activation, where the N‐terminal fragment of GSDME can synergise with Gasdermin D to enhance the efficiency of pyroptosis [15]. This synergistic effect plays an important role in the inflammatory response and immune response. The C‐terminal fragment of Gasdermin E also has an auto‐inhibitory function, similar to Gasdermin D, to regulate pore formation and the process of pyroptosis.

## 3 Cross‐Regulation Mechanisms Between Ferroptosis and Pyroptosis: Molecular Basis and Pathological Associations

Although ferroptosis and pyroptosis have unique mechanisms, they form complex crosstalk through shared regulatory nodes and synergistic signalling networks. This interaction plays a crucial role in the progression of multi‐system diseases, and its core mechanisms can be divided into several aspects, with the relevant content summarised in Table 1.

**TABLE 1:** Cross‐regulation molecular mechanisms between ferroptosis and pyroptosis.

Upstream factors/cross‐regulated molecular nodes	Mechanism of regulating ferroptosis	Mechanism of regulating pyroptosis	References
ROS	Iron ions catalyse H_2_O_2_ → •OH, which attacks PUFAs→ lipid peroxidation↑; GPX4 ↓ → lipid peroxidation products↑. The biliverdin, bilirubin produced by HO‐1 → oxidative stress↓, but the Fe^2+^ generated by HO‐1 → cellular damage↑	NLRP3 inflammasome↑ → caspase‐1 cleavage of GSDMD↑ → pyroptosis; A positive feedback loop of ROS with mitochondrial dysfunction→ the inflammatory response↑	[8, 15, 16, 17]
Ca^2+^	PLA_2_↑ → release PUFAs→ lipid peroxidation↑; NOX activity↑ → ROS ↑, creating a vicious cycle	Mitochondrial Ca^2+^ overload →MPT↑ → releasing cytochrome c → caspase‐9↑ → caspase‐1↑ → pyroptosis↑	[18, 19]
Mitochondrial dysfunction	Fe^2+^within cells↑ → ROS↑ → lipid peroxidation↑ and GPX4↓; Complex I/IV ↓ → ATP synthesis↓ → AMPK/mTOR signalling↓ → calcium homeostasis↓	ROS↑↑ → NLRP3↑ → caspase‐1 cleavage of GSDMD↑ → membrane pores formation → pyroptosis; Releases cytochrome c into the cytoplasm→caspase‐9↑ → caspase‐1 ↑ → pyroptosis↑	[19, 20]
Mitophagy and Ferritin Degradation Activate the cGAS‐STING Pathway	Ferritinophagy→Fe^2+^ → ROS↑ → cGAS‐STING pathway↑; Mitophagy removes damaged mitochondria→ mtROS↓ → ferroptosis↓	Mitochondrial autophagy→ mtROS↓ → cGAS‐STING pathway↓ → NLRP3 ↓ → pyroptosis↓	[21, 22, 23, 24, 25]
GSDMD pore‐mediated metabolic reprogramming	System Xc^−^↓ → intracellular cystine↓ → GSH synthesis↓ → GPX4 activity↓ → ferroptosis↑; NF‐κB‐NOX4 axis↑ → ROS↑ and oxidative stress↑	GSDMD pore formation → K^+^ efflux↑ → NLRP3‐caspase‐1 pathway↑ → inflammatory response↑	[1], [26, 27, 28, 29]
Caspase 1/3 Cross‐activation	caspase‐1↓ → SLC7A11↑ → GSH↑ and GPX4↑ → PUFA metabolism↓ → ferroptosis↓; caspase‐1↑ → cleave GSDMD→membrane pores formation→K^+^ efflux↑ → ferroptosis↑; caspase‐1↑ → NOX4↑ → ROS↑ → oxidative stress↑ → ferroptosis↑	ROS → caspase‐3 (through TOM20 and PARP1)↑→, cleaving GSDME→pyroptosis↑; caspase‐1↑ → cleave GSDMD→membrane pores formation→ pyroptosis ↑and the release of inflammatory factors↑	[7], [30, 31, 32, 33, 34]
Caspase‐8	SLC7A11 stability↓→, GSH synthesis↓ → ferroptosis↑	Cleaving GSDMD and GSDMC↑ → pyroptosis↑ → NLRP3 inflammasome↑ and release IL‐1β↑	[2], [35, 36]
The bridging function of autophagy	Degradation of ferritin→ free iron ions in cells↑ → ferroptosis↑; Mitochondrial autophagy→mtROS↓ → ferroptosis↓	Clearing damaged mitochondria → mtROS ↓ → NLRP3↓ → pyroptosis↓; LMP↑ → the release of CTSB and CTSD↑ → NLRP3↑ → pyroptosis↑	[37, 38, 39, 40, 41, 42, 43, 44, 45, 46, 47, 48, 49, 50, 51, 52, 53, 54, 55, 56, 57, 58, 59, 60, 61, 62, 63]
p53/STAT3/NRF2 Axis	p53 → SLC7A11↓ → GSH↓ → GPX4 ↓ → ferroptosis↑; p53 → ferroptosis↑ by GLS2 and TfR1↑; STAT3 → ferroptosis↑ by inflammatory factors↑; NRF2 → ferroptosis↓ by HO‐1 and NQO1↑	p53 → GSDME↑ and the NLRP3 ↑ → the cleavage of GSDMD↑ and the release of IL‐1β↑ → pyroptosis↑; The impact of STAT3 on pyroptosis varies across different diseases and models; NRF2 → NLRP3↓ → pyroptosis↓	[64, 65, 66, 67, 68, 69, 70, 71, 72]
HMGCR‐BRCC36 Axis	BRCC36 inhibits ferroptosis by deubiquitinating HMGCR, while HMGCR synthesises metabolites (such as coenzyme Q10 and GPX4) through the MVA pathway to suppress ferroptosis	BRCC36 promotes cell pyroptosis by deubiquitinating HMGCR. Under conditions that induce cell pyroptosis, HMGCR translocates to the endoplasmic reticulum and upregulates the expression of NLRP3 inflammasome and GSDMD	[6, 73]

### 3.1 Shared and Interacting Upstream Trigger Factors

#### 3.1.1 The Pivotal Role of Reactive Oxygen Species (ROS)

**FIGURE 1:**
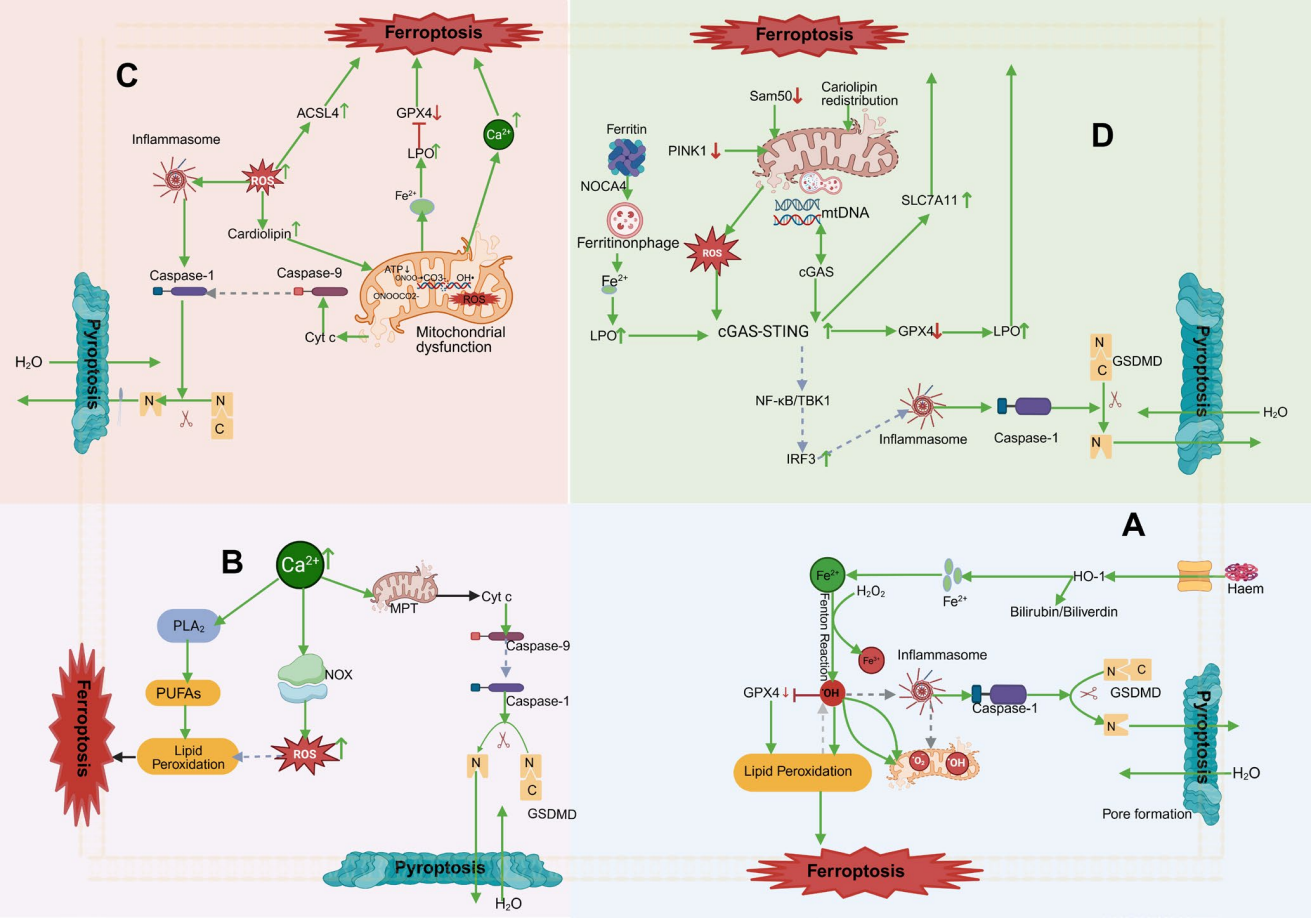
Shared and interacting upstream trigger factors in ferroptosis and pyroptosis. (A) ROS; (B) Ca^2+^; (C) Mitochondrial dysfunction; (D) cGAS‐ STING pathway. Green →, direct activation; grey ⇢, indirect activation; red ⊣, direct inhibition; red ‐ ‐ ‐ ⊣, indirect inhibition; green ↑, upregulation; red ↓, downregulation. Image created with BioRender.

As shown in Figure 1, during ferroptosis, intracellular iron catalyses the conversion of hydrogen peroxide (H_2_O_2_) into highly reactive hydroxyl radicals (˙OH) through the Fenton reaction [8]. These radicals attack PUFAs in the cell membrane, triggering lipid peroxidation. Meanwhile, the activity of GPX4 is inhibited during ferroptosis, further exacerbating the accumulation of lipid peroxidation products. This leads to the collapse of the cellular antioxidant system, ultimately causing cell membrane rupture and cell death [8].

However, the regulation of ROS by heme oxygenase‐1 (HO‐1) is closely related to ROS‐mediated ferroptosis. HO‐1 is a key stress‐response enzyme that degrades heme to produce biliverdin, carbon monoxide (CO), and ferrous ions (Fe^2+^). Biliverdin and bilirubin have significant antioxidant properties, effectively neutralising ROS and protecting cells from oxidative damage. However, Fe^2+^ reacts with H_2_O_2_ through the Fenton reaction to generate •OH, thereby increasing ROS production and exacerbating cellular damage [21]. This dual‐ action indicates that the function of HO‐1 within cells is Janus‐faced: on the one hand, its antioxidant products (biliverdin and bilirubin) can mitigate oxidative stress; on the other hand, the pro‐ oxidant effect of Fe^2+^ may exacerbate cellular damage. Therefore, the specific role of HO‐1 depends on the balance of the types, concentrations, and interactions of its degradation products [21].

Recent work provides direct proof that the HO‐1–Fe^2+^ axis acts as a shared trigger of ferroptosis and pyroptosis: Chen et al. [74] showed that hepatocyte‐specific loss of Sam50 elevates HO‐1 3.2‐fold, increases free Fe^2+^ by 2.4‐fold (FerroOrange), lowers GPX4 38%, raises lipid ROS 2.1‐fold and activates NLRP3–caspase‐1 with more IL‐1β release, all of which are reversed by HO‐1 siRNA or the Fe^2+^ chelator deferiprone; Fernand M et al. [16] extended this to LPS‐stimulated aged microglia, where adeno‐HO‐1 raises Fe^2+^ 1.9‐fold, drops GPX4 45% and boosts caspase‐1 p20 and GSDMD‐N, while deferiprone restores GPX4 and blocks NLRP3 in transgenic mice displaying memory deficits. Collectively, these data establish HO‐1‐derived Fe^2+^—rather than its antioxidant metabolites—as a core molecular node bridging ferroptotic and pyroptotic death.

Under pathological conditions such as inflammatory responses and ischemia‐reperfusion injury, the expression of HO‐1 is upregulated to enhance the cells' antioxidant capacity. However, the pro‐oxidant effect of Fe^2+^ may exacerbate cellular damage in some cases. Thus, the function of HO‐1 needs to be finely regulated to maintain the balance between its antioxidant and pro‐oxidant effects, thereby protecting cellular homeostasis and survival [21].

During pyroptosis activation, ROS act as damage‐associated molecular patterns (DAMPs) to activate the NLRP3 inflammasome, which promotes the cleavage of gasdermin D (GSDMD) by caspase‐1, thereby triggering pyroptosis [17]. Additionally, ROS form a positive feedback loop with mitochondrial dysfunction, further exacerbating the inflammatory response. Meanwhile, the activation of NLRP3 increases ROS levels, which in turn further exacerbate lipid peroxidation and the transmission of inflammatory signals [17].

The ROS‐NLRP3 positive feedback loop amplifies both types of cell death. Chen et al. [18] elaborated on this mechanism: the activation of NLRP3 leads to mitochondrial dysfunction, which causes the mitochondria to release more ROS. These ROS further attack PUFAs in the cell membrane, exacerbating lipid peroxidation. At the same time, the accumulation of ROS also promotes further activation of the NLRP3 inflammasome, forming a positive feedback loop. This positive feedback loop not only enhances the synergistic effect of ferroptosis and pyroptosis but may also exacerbate inflammatory responses and tissue damage under various pathological conditions. This complex interplay mechanism provides an important theoretical basis for understanding disease mechanisms and developing new therapeutic strategies.

#### 3.1.2 Calcium Ion (Ca^2+^) Dyshomeostasis

As shown in Figure 1, Calcium ion (Ca^2+^) dyshomeostasis plays a significant role in ferroptosis. On the one hand, calcium ions activate phospholipase A_2_ (PLA_2_), which releases PUFAs. These PUFAs, when attacked by ROS, exacerbate lipid peroxidation and drive the occurrence of ferroptosis [19]. On the other hand, Ca^2+^ can also regulate the activity of NADPH oxidase (NOX), promoting the generation of ROS and forming a vicious cycle that ultimately leads to ferroptosis [19].

Additionally, mitochondrial Ca^2+^ overload is one of the key factors that trigger pyroptosis. When intracellular Ca^2+^ concentrations are excessively high, mitochondria absorb too many Ca^2+^, leading to the occurrence of mitochondrial permeability transition (MPT), which in turn releases cytochrome c [20]. The release of cytochrome c is a crucial event in the apoptotic pathway; it can activate caspase‐9, initiating the apoptotic cascade [20]. The activation of caspase‐9 indirectly promotes the activation of caspase‐1, which cleaves GSDMD to form membrane pores. This results in cell swelling and rupture, ultimately triggering pyroptosis [20]. Therefore, calcium ion dyshomeostasis drives the occurrence of pyroptosis by affecting mitochondrial function and the caspase cascade.

#### 3.1.3 The Central Role of Mitochondrial Dysfunction

Mitochondrial dysfunction induces pyroptosis and ferroptosis through multiple pathways. The main manifestations include dysfunction of the tricarboxylic acid cycle (TCA), oxidative phosphorylation (OXPHOS), and the electron transport chain (ETC), as well as the disruption of Ca^2+^ homeostasis. Mitochondrial dysfunction leads to a significant increase in ROS, which activates the NLRP3 inflammasome, thereby activating caspase‐1 to cleave GSDMD and form cell membrane pores, inducing pyroptosis [20, 22]. Meanwhile, the release of cytochrome c from mitochondria into the cytoplasm activates caspase‐9, which in turn indirectly promotes the activation of caspase‐1, further exacerbating pyroptosis [20, 22].

Moreover, mitochondrial dysfunction leads to the accumulation of iron ions within cells. Iron ions catalyse lipid peroxidation through the Fenton reaction, compromising the integrity of the cell membrane and inactivating the GPx4 antioxidant system, ultimately triggering ferroptosis [20, 22]. Dysfunction of mitochondrial complexes I/IV impairs ATP synthesis, leading to AMPK/mTOR signalling imbalance and calcium homeostasis disruption. Lipid peroxidation products oxidise mitochondrial cardiolipin, and excessive mitochondrial ROS production activates Acyl‐CoA Synthetase Long‐Chain Family Member 4 (ACSL4), amplifying ferroptosis susceptibility and forming a self‐reinforcing damage loop [20]. This dual‐damage mechanism makes mitochondrial dysfunction a key regulatory node in various pathological conditions.

#### 3.1.4 Mitophagy and Ferritin Degradation Activate the cGAS‐STING Pathway

Mitophagy and ferritin degradation are crucial for maintaining cellular homeostasis by clearing damaged mitochondria and regulating iron metabolism. However, when these processes are impaired, mitochondrial DNA (mtDNA) leakage can activate the cGAS‐STING pathway, leading to inflammation and cellular damage, as detailed in several previous studies, as shown in Figure 1.

Chen et al. [74] showed that the absence of the mitochondrial outer membrane protein Sam50 in hepatocytes disrupts mitochondrial membrane integrity. Cardiolipin redistribution and aggregation alter mitochondrial structure, causing mtDNA release into the cytoplasm. This activates the cGAS‐STING pathway, triggering inflammation and hepatocyte injury. Zhou et al. [23] suggested that mitochondrial kinase PINK1 promotes mitophagy to prevent mtDNA leakage. PINK1 dysfunction leads to damaged mitochondria accumulation, mtDNA release, and cGAS‐STING pathway activation, causing cardiomyocyte injury and cardiac hypertrophy.

Li et al. [24] revealed that NCOA4‐mediated ferritin degradation releases iron ions, which catalyse lipid peroxidation via the Fenton reaction. This damages the cell membrane and activates the cGAS‐STING pathway. cGAS recognises cytoplasmic DNA (e.g., mtDNA), synthesises cGAMP and activates STING, leading to NF‐κB activation and cellular senescence. These studies highlight that mitophagy and ferritin degradation are essential for maintaining mitochondrial function and cellular homeostasis. Mitophagy defects exacerbate ferritin degradation, iron release, and mtDNA leakage, amplifying cGAS‐STING pathway activation and inflammation.

The cGAS‐STING pathway also plays a key role in pyroptosis. Under normal conditions, mtDNA is confined within mitochondria, but it can leak into the cytoplasm upon cellular damage or stress. cGAS binds to mtDNA, producing cGAMP, which activates STING. This triggers downstream signalling, including TBK1 and IRF3 activation, promoting type I interferon production and NLRP3 inflammasome assembly. Activated caspase‐1 cleaves GSDMD, forming pores in the cell membrane and inducing pyroptosis [25]. Oxidative stress can also activate the cGAS‐STING pathway by damaging mtDNA, leading to pyroptosis via NLRP3 inflammasome signalling [75].

Chen et al. [18] further revealed the cGAS‐STING pathway's role in ferroptosis. STING binds to GPX4, promoting its degradation and weakening cellular defence against lipid peroxidation. STING also regulates iron‐metabolism genes like SLC7A11, modulating ferroptosis sensitivity. This provides new insights into ferroptosis mechanisms and potential therapeutic targets for related diseases.

Recent structural‐function studies [26] reveal that activated STING directly engages GPX4 through hydrogen bonding between Thr267 of STING and Asn146 of GPX4, nucleating a STING–GPX4 complex that routes GPX4 to the autophagy‐lysosome pathway for degradation. Disrupting this interface—GPX4‐N146A or STING‐T267A—abolishes the interaction, restores GPX4 protein, suppresses lipid‐ROS accumulation, and halts ferroptosis. In vivo, either pharmacological blockade with the STING antagonist H‐151 or cardiomyocyte‐specific STING knockout preserves GPX4 levels and reduces infarct size in ischaemia–reperfusion mice, confirming the druggability and pathological relevance of the axis. Thus, the cGAS‐STING pathway propagates oxidative cell death via the precise cascade “mtDNA‐cGAMP‐STING(T267) → GPX4(N146) binding and degradation → ferroptosis amplification,” offering a structure‐defined target for multi‐pronged therapeutic intervention.

### 3.2 Direct Cross—Regulation of Molecular Nodes

#### 3.2.1 GSDMD Pore—Mediated Metabolic Reprogramming and Its Role in Cell Death

**FIGURE 2:**
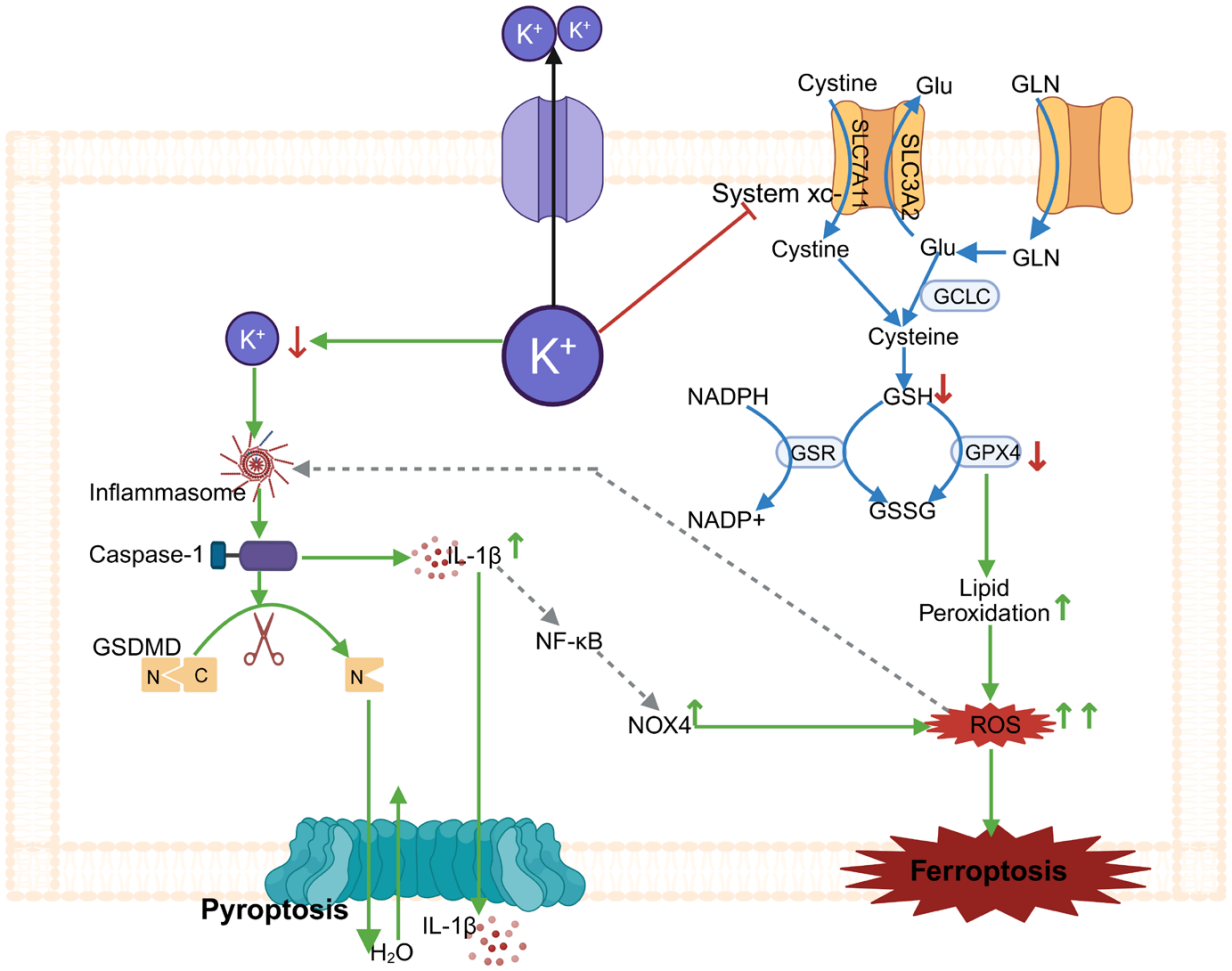
The role of GSDMD pore in pyroptosis‐ferroptosis crosstalk. Green →, direct activation; grey ⇢, indirect activation; red ⊣, direct inhibition; red ‐ ‐ ‐ ⊣, indirect inhibition; green ↑, upregulation; red ↓, downregulation. Image created with BioRender.

As shown in Figure 2, during pyroptosis, the formation of GSDMD pores leads to the efflux of intracellular potassium ions (K^+^). This process not only activates the non‐canonical inflammasome pathway of NLRP3‐caspase‐1, thereby causing the release of IL‐1β and amplifying the inflammatory response [26], but also triggers a series of metabolic changes by inhibiting the cystine/glutamate antiporter (System Xc^−^) [1]. Inhibition of System Xc^−^ results in decreased intracellular cystine levels, which in turn reduces the synthesis of GSH and the activity of GPX4, preventing effective clearance of lipid peroxides and ultimately increasing cellular sensitivity to ferroptosis [1, 28].

Moreover, inflammatory cytokines such as IL‐1β released in the early stages of pyroptosis further upregulate the expression of NADPH oxidase 4 (NOX4) via the NF‐κB signalling pathway, enhancing the generation of ROS [29, 30]. The accumulation of ROS not only exacerbates intracellular oxidative stress but also sets the stage for subsequent ferroptosis. Activation of NOX4 and increased ROS further weaken the cell's antioxidant defences, making it more susceptible to ferroptosis induction [30].

#### 3.2.2 Caspase Cross‐Activation Mechanisms Between Pyroptosis and Ferroptosis

**FIGURE 3:**
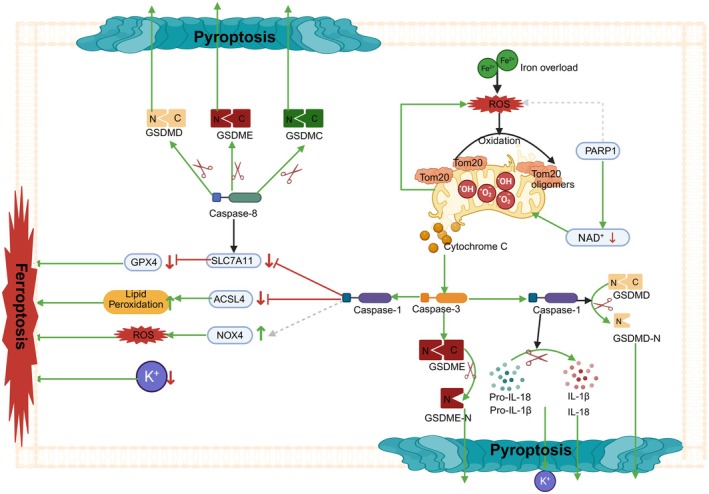
Caspase cross‐activation mechanisms between pyroptosis and ferroptosis. Green →, direct activation; grey ⇢, indirect activation; red ⊣, direct inhibition; red ‐ ‐ ‐ ⊣, indirect inhibition; green ↑, upregulation; red ↓, downregulation. Image created with BioRender.

Pyroptosis and ferroptosis are two important forms of programmed cell death, and there are complex interactions and cross‐signalling pathways between them. Recent studies have revealed the caspase cross‐activation mechanisms between pyroptosis and ferroptosis, as shown in Figure 3.

##### 3.2.2.1 Mechanism of ROS‐Activated Caspase‐3 During Ferroptosis

Research has shown that during ferroptosis, ROS signals induced by iron can be sensed by the mitochondrial outer membrane translocase 20 (TOM20). When intracellular ROS levels increase, TOM20 initiates a series of downstream signalling pathways, which in turn activate caspase‐3. Activated caspase‐3 can cleave GSDME, leading to the formation of cell membrane pores and thus triggering pyroptosis [7].

Moreover, ROS can also activate the DNA repair enzyme poly(ADP‐ribose) polymerase 1 (PARP1). Activation of PARP1 leads to a decrease in intracellular NAD^+^ levels, which in turn affects mitochondrial function and cellular energy metabolism. This metabolic disorder further exacerbates intracellular oxidative stress, ultimately triggering pyroptosis by activating caspase‐3 to cleave GSDME [31].

##### 3.2.2.2 Mechanism of Pyroptosis‐ Related Caspase‐ 1 Activation and Lipid Peroxidation

During pyroptosis, the activation of caspase‐1 can cleave GSDMD, leading to the formation of cell membrane pores, which not only promotes the release of inflammatory factors but also leads to the imbalance of intracellular ion homeostasis, especially the efflux of K^+^ [32]. As previously mentioned, the efflux of K^+^ further increases cellular sensitivity to ferroptosis [1, 28].

In addition, the activation of caspase‐1 can also upregulate the expression of NOX4 via the NF‐κB signalling pathway, enhancing the generation of ROS [33]. Activation of NOX4 and increased ROS further weaken the cell's antioxidant defence capabilities, making it more susceptible to ferroptosis induction [34]. Interestingly, inhibition of caspase‐1 can increase intracellular GSH and GPX4 levels by upregulating the expression of SLC7A11. GPX4 prevents lipid peroxidation by reducing lipid peroxides, thereby inhibiting ferroptosis. Moreover, inhibition of caspase‐1 can also reduce the metabolism of PUFAs by downregulating the expression of ACSL4, lowering cellular sensitivity to ferroptosis [35].

It is worth noting that the spatiotemporal synergistic effects between pyroptosis and ferroptosis have also attracted widespread attention from scholars. Inflammatory cytokines released in the early stages of pyroptosis (such as IL‐1β) further upregulate the expression of NOX4 via the NF‐κB signalling pathway, enhancing the generation of ROS [33]. The accumulation of ROS not only exacerbates intracellular oxidative stress but also creates conditions for subsequent ferroptosis. Activation of NOX4 and increased ROS further weaken the cell's antioxidant defence capabilities, making it more susceptible to ferroptosis induction [34].

In summary, there is a significant caspase cross‐activation mechanism between ferroptosis and pyroptosis. During ferroptosis, ROS activate caspase‐3 by activating TOM20 and PARP1, which in turn cleave GSDME to trigger pyroptosis; during pyroptosis, activation of caspase‐1 exacerbates cellular damage by enhancing lipid peroxidation, thereby affecting the ferroptosis process. This cross‐activation mechanism not only reveals the complex interplay between the two types of cell death but also provides new targets for developing therapeutic strategies for related diseases.

##### 3.2.2.3 The Balancing Role of Caspase‐8 in Pyroptosis and Ferroptosis

Previous studies have shown that caspase‐8 can cleave GSDMD and Gasdermin C (GSDMC), thereby redirecting the extrinsic apoptosis pathway toward pyroptosis, a process crucial for host defence against Yersinia infection. Specifically, caspase‐8 cleaves GSDMD at the D276 site, generating the active GSDMD‐NT fragment. This fragment targets the cell membrane and forms pores, leading to increased membrane permeability, K^+^ efflux, and activation of the NLRP3 inflammasome, which in turn promotes the maturation and release of IL‐1β and IL‐18 [2, 36]. Additionally, caspase‐8 can cleave GSDMC at the D365 or D240 site, triggering pyroptosis [2, 36]. In a DSS‐induced mouse model of colitis, Z‐DNA binding protein 1 (ZBP1), acting as a pattern recognition receptor, senses DNA damage or viral infection and is activated in DSS‐induced colitis, recruiting caspase‐8. Activated caspase‐8 cleaves GSDMC, generating the active GSDMC‐NT fragment, which targets the cell membrane to form pores, leading to increased membrane permeability, K^+^ efflux, and activation of the NLRP3 inflammasome, ultimately promoting the maturation and release of IL‐1β and IL‐18, triggering a strong inflammatory response that impairs mucosal repair, manifested as disruption of the mucosal barrier function and increased intestinal permeability [2]. Caspase‐8 has also been shown to cleave GSDME, generating the active GSDME‐NT fragment at the D240 site, triggering pyroptosis [2, 36].

Notably, recent literature has revealed the complex role of caspase‐8 in regulating ferroptosis and pyroptosis, particularly through its dynamic regulatory network by affecting the stability of SLC7A11 [37]. When caspase‐8 is activated, it can reduce the stability of SLC7A11 by cleaving it, thereby decreasing GSH synthesis and increasing cellular sensitivity to ferroptosis [37]. This mechanism indicates that caspase‐8 plays an important negative regulatory role in the process of ferroptosis. In summary, Caspase‐8 plays an important balancing role in pyroptosis and ferroptosis. By cleaving SLC7A11, Caspase‐8 can inhibit ferroptosis, thereby forming a dynamic regulatory network between pyroptosis and ferroptosis.

### 3.3 The Bridge Function of Autophagy

#### 3.3.1 The Regulatory Mechanism of Autophagy on Ferroptosis and Pyroptosis

**FIGURE 4:**
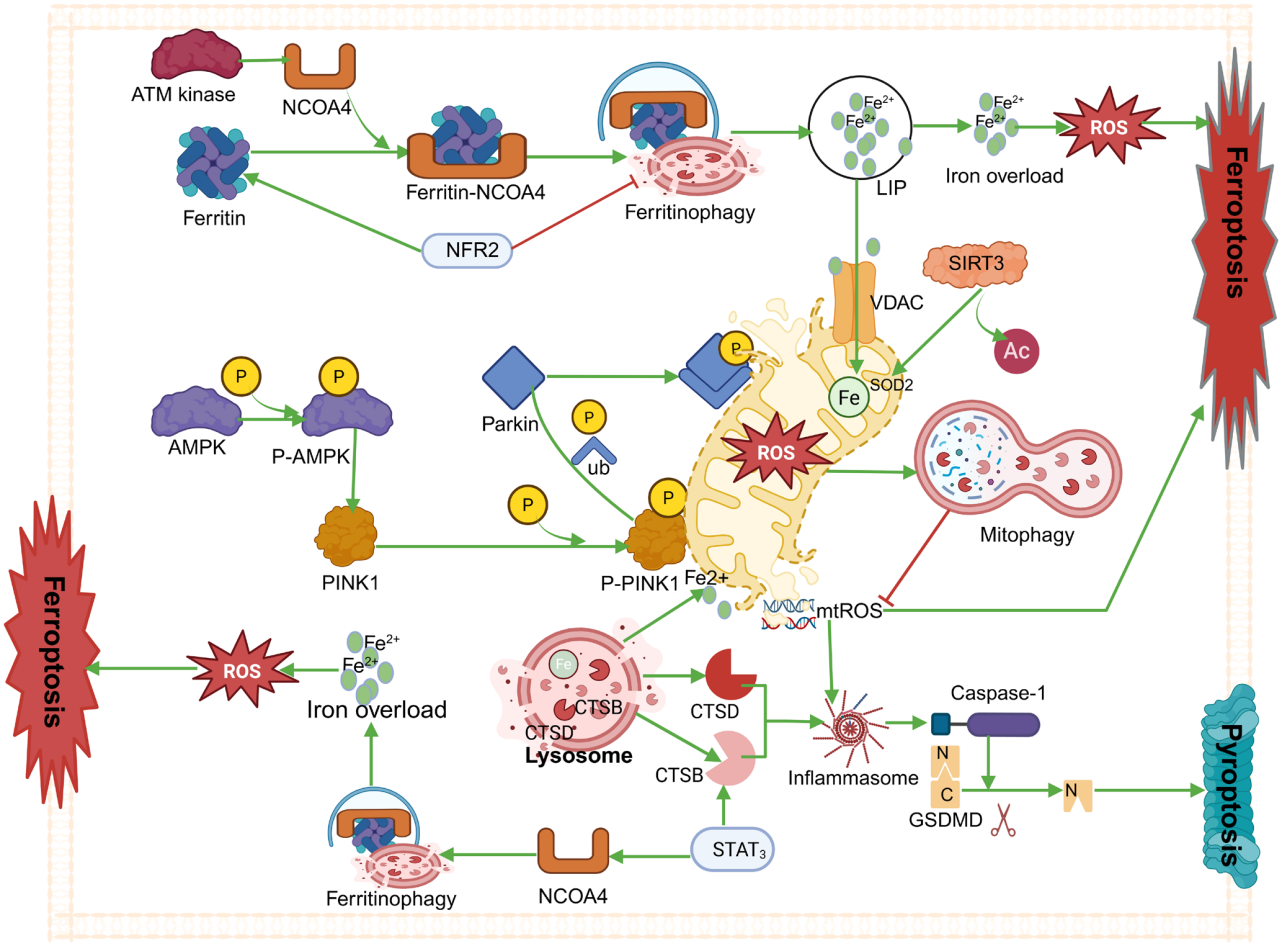
Autophagy serves as a bridge for the crosstalk between ferroptosis and pyroptosis. Green →, direct activation; grey ⇢, indirect activation; red ⊣, direct inhibition; red ‐ ‐ ‐ ⊣, indirect inhibition; green ↑, upregulation; red ↓, downregulation. Image created with BioRender.

In this section, we will elaborate on the regulatory mechanisms of autophagy on ferroptosis and pyroptosis, as well as the bridging role of mitophagy in the crosstalk between ferroptosis and pyroptosis, as shown in Figure 4.

##### 3.3.1.1 The Regulatory Mechanism of Autophagy on Ferroptosis

Ferritinophagy regulates intracellular iron ion levels through the selective degradation of ferritin. NCOA4‐mediated ferritinophagy forms cargo‐receptor complexes with ferritin, recruiting it into autophagosomes. After autophagy is completed, ferritin releases iron ions in lysosomes, increasing the level of labile iron ions (LIP) in the cytoplasm, which promotes ferroptosis [38, 39]. In this process, the Ser/Thr protein kinase ATM (ataxia telangiectasia mutated) and NRF2 play important roles. ATM kinase promotes the binding of NCOA4 (nuclear receptor coactivator 4) to ferritin by phosphorylating NCOA4, thereby inducing ferritinophagy and ferroptosis [40]. Additionally, NRF2 inhibits ferroptosis by upregulating the expression of ferritin and suppressing NCOA4‐mediated ferritinophagy. The inhibition of NRF2 promotes ferritinophagy and ferroptosis [41].

Mitophagy reduces intracellular ROS levels by clearing damaged mitochondria that produce excessive mtROS, thereby inhibiting ferroptosis. For example, PINK1‐parkin‐mediated mitophagy can reduce the production of mtROS, thus inhibiting ferroptosis [42, 43, 44]. The mitochondrial voltage‐dependent anion channel (VDAC) regulates the exchange of iron ions between mitochondria and the cytoplasm. VDAC‐mediated mitochondrial labile iron uptake reduces cytoplasmic LIP, but as mitochondrial labile iron accumulates and mitochondrial damage intensifies, it promotes ferroptosis [45, 46, 47].

##### 3.3.1.2 The Regulation of Pyroptosis by Autophagy

Autophagy reduces the production of mtROS by clearing damaged mitochondria, thereby inhibiting the activation of the NLRP3 inflammasome and alleviating pyroptosis. P62, an autophagy cargo receptor, recognises and binds to ubiquitinated mitochondrial proteins, recruiting them into autophagosomes for degradation. Excessive mtROS produced by damaged mitochondria can trigger the assembly and activation of the NLRP3 inflammasome, leading to the activation of caspase‐1 and the cleavage of GSDMD, ultimately inducing pyroptosis [48, 49, 50]. By clearing these damaged mitochondria, autophagy can effectively reduce the production of mtROS, thereby inhibiting the activation of the NLRP3 inflammasome and alleviating pyroptosis.

The PINK1/parkin pathway is one of the primary regulatory mechanisms of mitophagy. When mitochondria are damaged, the level of PINK1 protein on their outer membrane increases, recruiting Parkin protein to the mitochondrial surface. As an E3 ubiquitin ligase (E3) ubiquitin ligase, Parkin can ubiquitinate mitochondrial‐associated proteins, tagging damaged mitochondria for recognition and engulfment by autophagosomes, and ultimately degradation via lysosomes. This process clears damaged mitochondria and reduces the production of mtROS. With the decreased level of mtROS, the activation of the NLRP3 inflammasome is inhibited, thereby preventing the oligomerisation of ASC and the activation of caspase‐1, and consequently blocking the initiation of the pyroptosis‐related pathway and inhibiting pyroptosis [51]. For instance, quercetin promotes PINK1/parkin‐mediated mitophagy to reduce mtROS and NF‐κB activation, thereby inhibiting LPS‐induced pyroptosis in microglia [52]. Moreover, mitophagy exerts a negative feedback on mtROS‐mediated inflammasome activation by reducing mtROS production, maintaining the homeostasis of intracellular ROS levels, and preventing excessive inflammatory responses, thus inhibiting pyroptosis [53].

In an in vitro model of hypoxic–ischemic brain injury, exosomes derived from mesenchymal stem cells enhance autophagy, which clears damaged mitochondria, reduces mtROS production, and inhibits the activation of the NLRP3 inflammasome, thereby suppressing pyroptosis and protecting microglial cells [54]. PINK1‐mediated autophagy exerts a protective role in postoperative cognitive dysfunction by clearing damaged mitochondria, reducing mtROS production, and inhibiting caspase‐3/GSDME‐dependent pyroptosis, thereby alleviating postoperative cognitive deficits [55].

In this process, many key regulators play important roles. For example, in human macrophages, Sirtuin 3 (SIRT3) activates mitochondrial antioxidant enzymes, such as superoxide dismutase 2 (SOD2), through deacetylation, enhancing the mitochondria's antioxidant capacity and reducing ROS production. This action of SIRT3 effectively inhibits the over‐activation of the NLRP3 inflammasome, thereby suppressing the occurrence of pyroptosis [56]. Parkin can also inhibit the activation of NF‐κB by upregulating the expression of the anti‐apoptotic protein A20, reducing the production of inflammatory factors, and thus inhibiting the activation of the NLRP3 inflammasome, ultimately achieving the goal of suppressing pyroptosis [57].

#### 3.3.2 Autophagy Serves as a Bridge for the Crosstalk Between Ferroptosis and Pyroptosis

Autophagy plays a crucial regulatory role in ferroptosis and pyroptosis through various mechanisms. For example, increased lysosomal membrane permeability leads to the release of lysosomal contents (such as iron ions, Cathepsin B [CTSB], and Cathepsin D [CTSD]) into the cytoplasm, which activates the NLRP3 inflammasome and induces both pyroptosis and ferroptosis [58, 59, 60]. The release of iron ions from lysosomes increases the level of the labile iron pool (LIP) in the cytoplasm, thereby promoting ferroptosis. Meanwhile, the release of CTSB and CTSD can activate the NLRP3 inflammasome, triggering pyroptosis [58, 59, 60]. After entering the nucleus, CTSB can cause DNA damage, activate the cGAS‐STING pathway, and subsequently induce autophagy and pyroptosis [61, 62]. Additionally, STAT3 upregulates the expression of CTSB and Nuclear Receptor Coactivator 4 (NCOA4), enhancing autophagy and ferritinophagy. The increase in CTSB can directly activate the NLRP3 inflammasome, while the upregulation of NCOA4 promotes ferritinophagy, increases the level of intracellular free iron ions, and thereby promotes ferroptosis [62, 63, 64]. Moreover, the MEK–ERK pathway, by phosphorylating STAT3, further upregulates the expression of CTSB and NCOA4, thereby enhancing ferritinophagy and ferroptosis [63].

### 3.4 Synergy and Antagonism in Transcriptional Regulatory Networks

#### 3.4.1 Regulatory Role of the p53/STAT3/NRF2 Axis Between Ferroptosis and Pyroptosis

**FIGURE 5:**
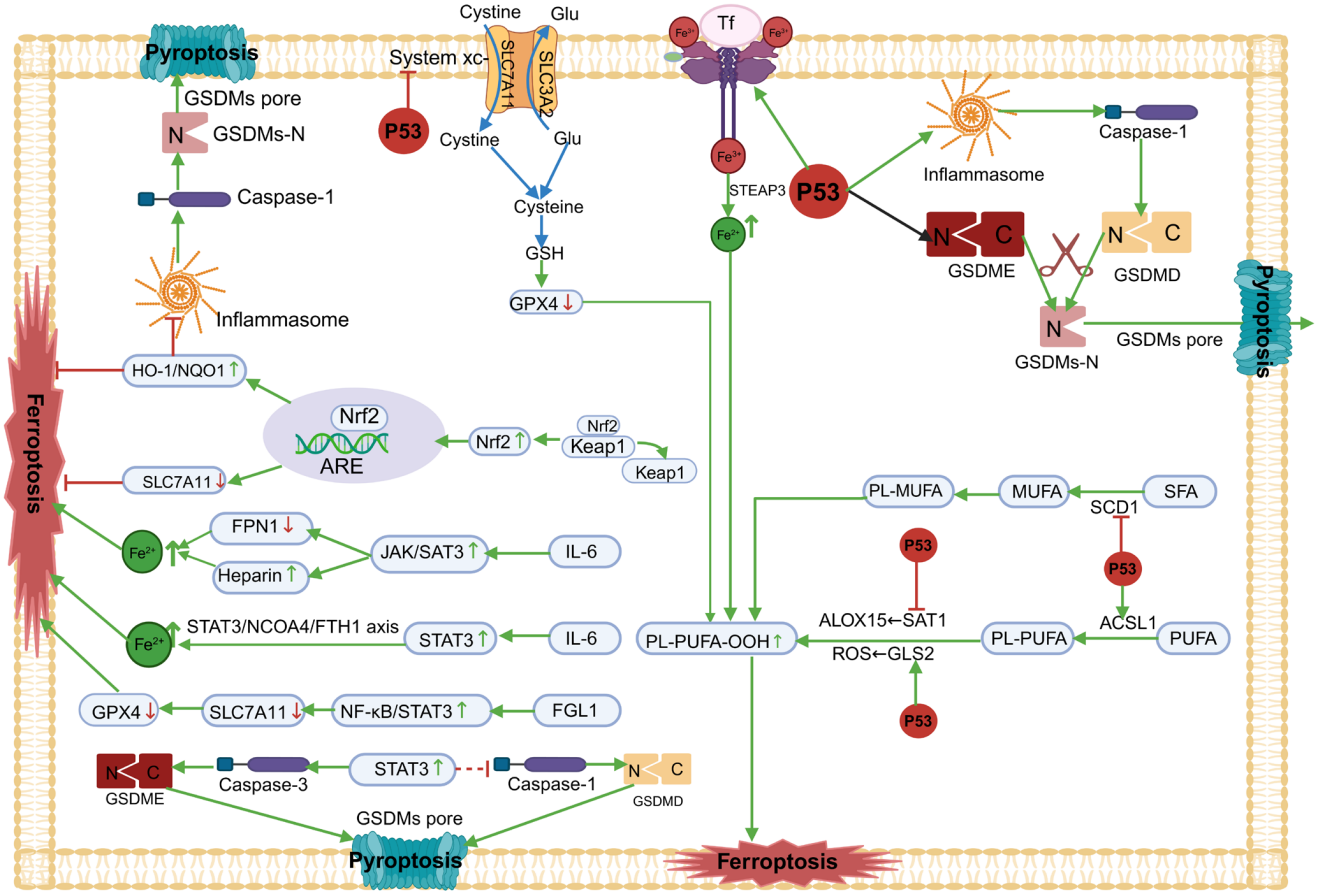
Regulatory role of the p53/STAT3/NRF2 axis between ferroptosis and pyroptosis. Green →, direct activation; grey ⇢, indirect activation; red ⊣, direct inhibition; red ‐ ‐ ‐ ⊣, indirect inhibition; green ↑, upregulation; red ↓, downregulation. Image created with BioRender.

Cell death is a complex process that is finely regulated by multiple signalling pathways. In recent years, it has been found that p53, Nrf2, and STAT3 play key roles in the pathways of ferroptosis and pyroptosis. These transcription factors influence cell survival and death by regulating specific gene expression and signalling pathways, as shown in Figure 5.

##### 3.4.1.1 Regulatory Role of p53 in Cell Death

p53 is an important tumour suppressor that is widely involved in cell cycle regulation, DNA repair, and cell death. The study by Liu et al. [65] summarised the regulatory roles of p53 in ferroptosis and pyroptosis. The specific mechanisms are as follows: First, p53 inhibits the expression of SLC7A11, thereby reducing cystine uptake and subsequently decreasing the level of GSH, which weakens the activity of GPX4 and leads to the accumulation of lipid peroxides, thus inducing ferroptosis. In addition, p53 can promote ferroptosis through multiple pathways, such as regulating lipid metabolism (e.g., upregulating ACSL4 or inhibiting Stearoyl‐CoA Desaturase 1 [SCD1] expression), activating Glutaminase 2 (GLS2) to enhance ROS generation, activating TfR1 to regulate iron metabolism, and inducing Spermidine/spermine N1‐acetyltransferase 1(SAT1) expression to affect polyamine metabolism. Notably, p53 can also inhibit ferroptosis under certain circumstances by regulating anti‐ferroptotic systems (e.g., inhibiting Phosphoglycerate Dehydrogenase [PHGDH] or Cystathionine‐beta‐synthase [CBS expression] or activating iPLA2β), indicating that p53 has a complex dual role in ferroptosis regulation, with the ultimate effect depending on cell type and stress conditions [65]. Moreover, p53 can activate GSDME and the NLRP3 inflammasome, promote the cleavage of GSDMD, release IL‐1β, and thus facilitate pyroptosis and immune response. Upon DNA damage, p53 can dominate the switch of cell death modes, selectively activating ferroptosis or pyroptosis [65].

##### 3.4.1.2 Regulatory Role of Nrf2 in Cell Death

Nrf2 is a key antioxidant transcription factor that can upregulate the expression of multiple antioxidant genes, thereby enhancing the cellular antioxidant capacity. The study by Chen et al. [66] discussed in detail the role of Nrf2 in ferroptosis and pyroptosis. Nrf2 inhibits ferroptosis by upregulating antioxidant genes such as HO‐1 and NAD(P)H Quinone Dehydrogenase 1 (NQO1), which in turn scavenge intracellular ROS and reduce lipid peroxidation [66]. Additionally, Nrf2 indirectly inhibits the activation of the NLRP3 inflammasome by regulating the expression of antioxidant genes HO‐1 and NQO1, thereby alleviating pyroptosis [66].

##### 3.4.1.3 Regulatory Role of STAT3 in Cell Death

Signal transducer and activator of transcription 3 (STAT3) has dual roles in cell survival and death. Xie et al. [67] reported that in chronic stress‐induced duodenal injury, IL‐6 activates the JAK2/STAT3 axis, inhibiting Ferroportin 1 (FPN1) expression, increasing hepcidin production, and disrupting intracellular iron homeostasis to promote ferroptosis. Similarly, in obesity‐induced cardiac injury, IL‐6 activates STAT3 via the STAT3/NCOA4/FTH1 axis to induce ferroptosis. STAT3 also regulates inflammatory factor expression. In Crohn's disease, FGL1 activates the NF‐κB/STAT3 axis to inhibit SLC7A11 and GPX4, promoting intestinal epithelial cell ferroptosis [68].

Conversely, Li et al. [69] showed that vagus nerve stimulation (VNS) activates the α7nAchR, promoting STAT3 phosphorylation and activation. Activated STAT3 reduces NLRP3 inflammasome assembly and caspase‐1 maturation, inhibiting GSDMD cleavage and membrane pore formation, thereby alleviating pyroptosis. In anARDS model, VNS reduced pyroptotic cells in lung tissue by activating STAT3.

In tumours, STAT3 regulates pyroptosis differently [70]. In oesophageal squamous cell carcinoma (ESCC), STAT3β activates caspase‐3 and GSDME to trigger pyroptosis and enhance chemotherapeutic drug sensitivity [71]. In malignant melanoma, combined BRAF/MEK inhibition induces caspase‐3 activation and GSDME cleavage, with STAT3 potentially enhancing therapeutic effects by regulating inflammasome activation and pyroptosis‐related gene expression [72]. In acute myeloid leukaemia (AML), STAT3 affects leukaemia cell pyroptosis by regulating inflammasome activation and pyroptosis‐related gene expression, with GSDMD serving as a therapeutic sensitivity biomarker [71]. In colorectal cancer (CRC), STAT3 activation reduces inflammasome assembly and caspase‐1 maturation, inhibiting GSDMD cleavage and alleviating pyroptosis‐induced tissue damage [73].

In summary, p53, Nrf2 and STAT3 play complex regulatory roles in cell death pathways. p53 promotes ferroptosis and pyroptosis by inhibiting antioxidant pathways and activating inflammasomes; Nrf2 antagonises ferroptosis and alleviates pyroptosis by upregulating antioxidant genes; STAT3 promotes ferroptosis by regulating iron metabolism and the expression of inflammatory factors. However, its impact on pyroptosis varies in different diseases and models, and further research is needed.

#### 3.4.2 The HMGCR‐BRCC36 Axis as a Metabolic Reprogramming Hub

In recent years, the interaction between BRCA1/BRCA2‐containing complex subunit 36 (BRCC36) and 3‐Hydroxy‐3‐Methylglutaryl‐CoA Reductase (HMGCR) has garnered significant attention for its role in the regulatory mechanisms of ferroptosis and pyroptosis, as well as its applications in hepatocellular carcinoma (HCC). Wang et al. [6] revealed that BRCC36 regulates the interplay between ferroptosis and pyroptosis by deubiquitinating HMGCR. As a deubiquitinase, BRCC36 specifically removes K63 polyubiquitin chains from HMGCR, thereby stabilising the HMGCR protein. During ferroptosis, HMGCR is primarily localised in the mitochondria, where it inhibits ferroptosis by enhancing the synthesis of GPX4 and coenzyme Q10. However, upon treatment with pyroptosis inducers, HMGCR translocates to the endoplasmic reticulum, thereby promoting pyroptosis [6]. Additionally, the BRCC36 inhibitor thiolutin can effectively disrupt the interaction between BRCC36 and HMGCR, enhancing ferroptosis induced by the ferroptosis inducer (1S,3R)‐RSL3 (RSL3) and inhibiting the growth of HCC [6].

Hsu SK et al. [76] further explored the critical role of deubiquitinases (DUBs) in ferroptosis and pyroptosis and their potential as cancer intervention targets. The study indicated that BRCC36 regulates the balance between ferroptosis and pyroptosis by deubiquitinating HMGCR. During ferroptosis, HMGCR inhibits ferroptosis through the synthesis of metabolites in the mevalonate (MVA) pathway, such as coenzyme Q10 and GPX4; under pyroptosis‐inducing conditions, HMGCR translocates to the endoplasmic reticulum, promoting pyroptosis by upregulating the expression of the NLRP3 inflammasome and GSDMD [76].

These studies not only provide new mechanistic insights into the complex interplay between ferroptosis and pyroptosis but also offer potential molecular targets for the treatment of HCC. The mechanism by which BRCC36 regulates ferroptosis and pyroptosis through deubiquitination of HMGCR suggests that targeting BRCC36 could emerge as a novel cancer intervention strategy, offering a new direction for the precision treatment of HCC.

In summary, the NLRP3 inflammasome and ferroptosis play crucial roles in various diseases, with complex interactions. From cerebral ischemia–reperfusion injury to sepsis, the temporal and spatial mechanisms suggest that single‐factor interventions may be insufficient. Future research should focus on multi‐target synergistic strategies, such as combining NLRP3 inhibitors with ferroptosis regulators. The development of nanotechnology and immunomodulators also holds promise for disease treatment. A deeper understanding of these mechanisms can provide more precise and effective therapeutic strategies to improve patient outcomes.

## 4 Temporal and Spatial Synergy in Disease Models and Therapeutic Insights

### 4.1 Ischemia–Reperfusion Injury

In cerebral ischemia–reperfusion injury, NLRP3 inflammasome activation is a key factor in neuronal damage and release. Wu et al. [77] showed that the NLRP3 inflammasome is activated early in ischemia, βcleaving GSDMD and releasing IL‐1β, which triggers inflammation and neuronal pyroptosis. During reperfusion, ROS accumulation and GSH depletion dominate, leading to increased lipid ROS levels, decreased GSH/GSSG ratios, and increased iron content, inducing neuronal ferroptosis. The NLRP3 inflammasome inhibitor MCC950 reduces GSDMD cleavage and IL‐1β release, alleviating pyroptosis, and also regulates oxidative stress levels, reducing lipid ROS generation, increasing the GSH/GSSG ratio, and decreasing iron accumulation, thus mitigating ferroptosis. This multi‐target intervention mechanism indicates that MCC950 can effectively inhibit NLRP3 inflammasome‐mediated neuronal pyroptosis and ferroptosis, providing a new strategy for treating cerebral ischemia–reperfusion injury [77].

### 4.2 Infection and Sepsis

Lin et al. [78] investigated the roles of nanoscale immunomodulators in sepsis, highlighting that bacterial lipopolysaccharide (LPS) activates the TLR4 pathway, leading to NLRP3 inflammasome activation, GSDMD cleavage, membrane pore formation, and release of inflammatory cytokines (IL‐1β and IL‐18), triggering pyroptosis. LPS also downregulates hepcidin, increasing iron uptake and inducing ferroptosis. Intracellular iron catalyses lipid peroxidation, causing cell membrane damage and death.

Therapeutic strategies targeting these mechanisms include using drugs like Disulfiram to block GSDMD pore formation and alleviate pyroptosis [79, 80]. And supplementing with GSH precursors like N‐acetylcysteine (NAC) to enhance antioxidant capacity and relieve ferroptosis [81]. These multi‐target interventions can improve sepsis prognosis, though no reports yet exist on combining Disulfiram and NAC for sepsis treatment.

### 4.3 Tumour Subtypes

#### 4.3.1 Triple‐Negative Breast Cancer (TNBC)

In TNBC, the hypoxic microenvironment enhances HIF‐1α expression, which not only promotes cellular adaptation to low oxygen conditions but also exacerbates cancer progression by upregulating pro‐inflammatory cytokines such as IL‐1β. The role of IL‐1β in activating NLRP3, thereby driving pyroptotic cell death and amplifying inflammatory responses within the tumour microenvironment, has been highlighted [82, 83]. Additionally, HIF‐1α‐regulated pathways can augment iron accumulation and lipid peroxidation—hallmark features of ferroptosis. For instance, dimethylmalonate has been shown to induce ferroptosis via the SUCNR1/PI3K/HIF‐1α signalling cascade, resulting in the suppression of key antioxidant proteins, including SLC7A11 and GPX4, thereby increasing susceptibility to oxidative damage [84].

TNBC exhibits an “immune‐inflammatory” microenvironment, wherein highly infiltrated CD8^+^ T cells upregulate NOX4 expression via IFN‐γ, forming a ROS‐NLRP3 positive feedback loop that drives synergistic amplification between ferroptosis and pyroptosis [85].

Targeting these cell death pathways offers a promising therapeutic strategy against TNBC. The interplay between HIF‐1α and NLRP3 marks a potential therapeutic target for reducing inflammation and promoting tumour cell death. For example, studies on the anti‐cancer properties of coenzyme Q demonstrate its dual role in inhibiting HIF‐1α and blocking NLRP3 inflammasome activation. This not only alleviates cancer‐associated inflammation but also influences metabolic processes by shifting cancer cell metabolism from aerobic glycolysis toward mitochondrial oxidative phosphorylation, thereby creating a microenvironment unfavourable for tumour growth and metastasis [85].

#### 4.3.2 Intrahepatic Cholangiocarcinoma (iCCA)

In cholangiocarcinoma, ferroptosis is initiated by GPX4 inactivation, ACSL4‐mediated lipid peroxidation, and TFRC‐driven iron overload, with bile acid accumulation and the hypoxia/HIF‐1α/BNIP3 axis further exacerbating metabolic disorder [86], while pyroptosis depends on NLRP3 inflammasome activation, whereby caspase‐1 cleaves GSDMD to release IL‐1β/IL‐18, mediating inflammatory cell death induced by chemotherapeutic agents such as methotrexate [87]. Although existing literature has not directly elucidated the crosstalk mechanism between these pathways, oxidative lipids and free iron ions generated during ferroptosis can theoretically act as damage‐associated molecular patterns (DAMPs) to activate NLRP3, thereby converting metabolic collapse signals into a pyroptotic cascade. This crosstalk may display spatiotemporal heterogeneity (ferroptosis in the tumour core and pyroptosis at the infiltrating margin), where Li Zuyin et al. [88] recently revealed that the infiltrating margin harbours a unique ‘triad structure’ composed of SPP1+ macrophages, POSTN+ FAP+ fibroblasts, and endothelial cells that creates an immunosuppressive niche, potentially dampening pyroptotic surveillance while the core's immune desert phenotype favours ferroptosis evasion [86, 87], representing a key node for therapeutic resistance.

Unlike the immune‐inflammatory microenvironment of TNBC, iCCA's SPP1+ macrophage‐POSTN+ fibroblast‐endothelial cell triad structure suppresses pyroptosis surveillance through the following mechanisms: (1) SPP1‐CD44 interaction blocks caspase‐1 activation; (2) POSTN matrix physical barrier restricts CTL infiltration; (3) Endothelial cells highly express SLC40A1 (ferroportin) to export iron ions, reducing iron concentration in the tumour core. This leads to the accumulation of ferroptosis signals (lipid ROS) in the core region that cannot trigger NLRP3, forming a ‘ferroptosis‐without‐pyroptosis’ resistance pattern. This structure is not found in either TNBC or AD and represents a unique resistance node for iCCA.

Future therapeutic strategies employing ferroptosis inducers (erastin/RSL3) in combination with NLRP3 inhibitors (MCC950), or pyroptosis activators synergised with iron chelators, could break the vicious metabolic‐inflammatory cycle by reshaping the immune microenvironment, particularly by disrupting the SPP1‐CD44 interaction within this triad structure to restore death pathway sensitivity and overcome chemoresistance in CCA [86, 87].

### 4.4 Neurodegenerative Diseases

#### 4.4.1 Alzheimer's Disease (AD)

In Alzheimer's disease (AD), neuronal ferroptosis and microglial pyroptosis establish a pathological crosstalk through a metabolic‐inflammatory positive feedback loop: Aβ plaques induce iron overload and lipid peroxidation, driving neuronal ferroptosis [89], while oxidative products released from dying neurons serve as DAMPs to activate the microglial NLRP3 inflammasome, with TLR4 recognition triggering GSDMD‐mediated pyroptosis and IL‐1β release [90]; IL‐1β subsequently upregulates neuronal NOX4 expression and ferritinophagy, exacerbating ferroptosis and thereby creating a vicious cycle. This crosstalk mechanism suggests that combined targeting of ferroptosis (Ferrostatin‐1) and NLRP3 (MCC950) may yield synergistic therapeutic effects. Based on the aforementioned crosstalk mechanism, therapeutic strategies should bidirectionally interrupt this pathological loop: iron chelators and GPX4 activators can suppress neuronal ferroptosis [89], whereas NLRP3 inhibitors such as MCC950 or TLR4 antagonists can block microglial pyroptosis [90]; their combined use demonstrates synergistic effects in AD models, reducing Aβ deposition and improving cognition, thereby providing translational medical evidence for precision therapy targeting metabolic‐inflammatory crosstalk.

Its microenvironment exhibits distinct specificity. In AD brain tissue, Aβ plaques serve as an ‘iron reservoir’ that is selectively deposited around neurons, causing a regional Fe^3+^/Fe^2+^ ratio imbalance (distinct from the systemic iron overload in iCCA). This iron pool continuously releases Fe^2+^ via ferritinophagy, driving neuron‐specific ferroptosis. Meanwhile, activated microglia (rather than astrocytes) recognise oxidised phospholipids through TLR4, initiating GSDMD‐mediated pyroptosis and forming a unidirectional cycle of ‘neuronal death → glial activation → inflammatory spread’. This is fundamentally different from the bidirectional interaction of ‘cancer cell death → immune suppression’ in iCCA.

#### 4.4.2 Parkinson's Disease (PD)

In Parkinson's Disease (PD), ferroptosis is driven by dysregulation of transcription factors such as NRF2 and HIF‐1α, which downregulate SLC7A11/GPX4 and upregulate ACSL4, leading to iron accumulation and lipid peroxidation [91]. Pyroptosis, on the other hand, occurs through microglial NLRP3 inflammasome activation, which cleaves GSDMD to release IL‐1β/IL‐18 and mediates neuroinflammation [92].

The crosstalk mechanism between the two lies in the fact that oxidised phospholipids produced by neuronal ferroptosis act as DAMPs to activate NLRP3, initiating microglial pyroptosis and releasing IL‐1β, which further downregulates neuronal GPX4 expression and exacerbates susceptibility to ferroptosis, forming a vicious cycle. This metabolic‐inflammatory dual hit is particularly significant in Alzheimer's disease and ischemic brain injury, suggesting that combined targeting with iron chelators and NLRP3 inhibitors may produce synergistic neuroprotective effects [91, 92].

The substantia nigra pars compacta (SNpc) microenvironment in PD is characterised by mitochondrial dysfunction as its core feature (complex I deficiency), leading to local ATP depletion and accumulation of dopamine metabolites (DOPAL). These two factors synergistically inhibit GPX4 activity, rendering dopaminergic neurons highly sensitive to ferroptosis. In contrast, iCCA cholangiocytes gain resistance by upregulating GPX4 via PAX8‐AS1, whereas PD neurons cannot activate this pathway. This cell‐type specificity determines that the pyroptosis‐ferroptosis crosstalk in PD is primarily characterised by ‘neuroinflammation‐driven ferroptosis’ rather than bidirectional activation.

Based on the aforementioned crosstalk mechanism, therapeutic strategies should synergistically target both ferroptosis and pyroptosis: for ferroptosis inhibition [91], iron chelators can be used to reduce iron accumulation, NRF2 activators to restore SLC7A11/GPX4 expression, or ACSL4 inhibitors to block lipid peroxidation; for pyroptosis inhibition [92], NLRP3 inhibitors (such as MCC950), GSDMD blockers, or IL‐1β neutralizing antibodies can effectively alleviate microglia‐mediated neuroinflammation. The combined use of these approaches can synergistically break the vicious cycle of ‘neuronal ferroptosis → microglial pyroptosis → IL‐1β release → exacerbated ferroptosis,’ providing a precise dual‐intervention therapeutic regimen for conditions such as Alzheimer's disease and ischemic brain injury. The combined use of these strategies can synergistically disrupt the vicious cycle of ‘neuronal ferroptosis → microglial pyroptosis → IL‐1β release → exacerbation of ferroptosis’, providing a precise dual‐intervention therapeutic regimen for diseases such as Alzheimer's disease and ischemic brain injury.

In summary, the NLRP3 inflammasome and ferroptosis orchestrate complex pathological networks across diverse diseases, operating through spatiotemporal mechanisms that render single‐agent interventions inadequate. From cerebral ischemia–reperfusion injury—where NLRP3 activation precedes ferroptosis during reperfusion—to sepsis, where LPS simultaneously triggers GSDMD‐mediated pyroptosis and iron‐dependent ferroptosis, these pathways exhibit intricate crosstalk that amplifies tissue damage. In triple‐negative breast cancer and cholangiocarcinoma, HIF‐1α coordinates a dual metabolic‐inflammatory assault, while neurodegenerative diseases showcase a vicious cycle where neuronal ferroptosis fuels microglial pyroptosis, further exacerbating neuronal vulnerability. Collectively, these disease models underscore that therapeutic efficacy requires multi‐target strategies, such as combining NLRP3 inhibitors like MCC950 with ferroptosis modulators (erastin/RSL3, Ferrostatin‐1), iron chelators or GSDMD blockers. The emergence of nanoscale immunomodulators and dual‐function agents like coenzyme Q exemplifies the potential for integrated approaches that simultaneously disrupt inflammatory and metabolic cascades. Future research must prioritise understanding pathway‐specific temporal dynamics and tumour microenvironment heterogeneity to develop precision combination therapies, ultimately translating these mechanistic insights into improved clinical outcomes for patients with cancer, neurodegenerative, and ischemic diseases.

## 5 Drug Development Strategies Based on the Synergistic Regulation of Ferroptosis and Pyroptosis

In this section, we summarised the drugs based on the synergistic regulation of ferroptosis and pyroptosis, including small molecule inhibitors, metal complexes and photosensitizers, nanoparticle drug delivery systems, natural monomer compounds, and repurposing of old drugs (such as sorafenib), with the relevant content summarised in Table 2.

**TABLE 2:** Drug development strategies based on the synergistic regulation of ferroptosis and pyroptosis.

Strategy classification	Drug/System	Mechanism of action	Experimental model/drug usage	Key results	Advantages and challenges	References
Small molecule inhibitors	Thiolutin + Lovastatin	BRCC36 inhibition → HMGCR degradation → dual ferroptosis↑ & pyroptosis↓	Mouse HCC subcutaneous model (C57BL/6, male, 8 weeks) Thiolutin: 0.75 mg/kg, i.p., q3d × 6 doses (18 days) Vehicle: 5% DMSO +45% PEG300 + 2% Tween‐80 in PBS, pH 7.2 Purity: ≥ 98% (HPLC) Lovastatin: 5 mg/kg, oral gavage, daily	Tumour growth inhibition: 68% (combo) vs. 22% (mono); HMGCR↓ by 73%	Advantages: Dual pathway targeting Challenge: DUB selectivity; requires i.p. route	Wang et al. [6]
Metal complexes	Iridium(III) photosensitizer (IrC/IrF)	PDT → ROS↑ → KEAP1/NRF2 → HO‐1↑ → Fe^2+^↑ → ferroptosis; ROS → GSDME cleavage→ pyroptosis	Mouse breast cancer 4T1 bilateral model (BALB/c, female, 6–8 weeks) IrC/IrF: 5 mg/kg, intratumoral, once daily × 16 days Light: 425 nm, 20 mW/cm^2^, 15 min post‐injection Vehicle: 0.9% saline Purity: ≥ 95% (elemental analysis)	Primary tumour: 85% reduction; Lung metastasis: 90% reduction; ICD markers (CRT, HMGB1)↑	Advantages: Spatiotemporal control Challenge: Deep tissue penetration; metabolic clearance unknown	Zeng et al. [92]
Nanodrug Delivery System	Tf‐LipoMof@PL	pH‐responsive Fe^3+^ release → Fenton reaction → ferroptosis; H_2_O_2_ → NLRP3 → GSDMD cleavage→pyroptosis	Mouse 4T1 subcutaneous model (BALB/c, female, 7 weeks) Tf‐LipoMof@PL: 5 mg/kg, i.v., once every 2 days × 10 doses (21 days) Vehicle: PBS, pH 7.4 Purity: > 99% (DLS & TEM verification) Stability: < 5% leakage over 48 h	Tumour Fe/ROS↑ 4.2‐fold; Systemic CD8^+^ T cell activation↑ 3.1‐fold	Advantages: Dual synergistic effect Challenge: Long‐term biodistribution; GMP manufacturing	Xu et al. [93]
	Thunder God Vine Tetrandrine Nano Preparation (FA‐MOF)	Fe^3+^+TPL → ROS↑ → ferroptosis; TPL → Nrf2↓ → NLRP3↑ → pyroptosis	C57BL/6 melanoma subcutaneous & lung metastasis model (male, 8 weeks) TPL: 0.6 mg/kg, i.v., on days 0, 3, 6 (3 doses total) Vehicle: 10% ethanol +40% PEG400 + 5% glucose, pH 6.5 Purity: ≥ 99% (LC–MS)	Tumour suppression: 80%; Enhanced anti‐PD1 response (combo)	Advantages: FA targeting Challenge: TPL systemic toxicity; narrow therapeutic window	Wang et al. [94]
Natural compounds	Ginsenoside Rh3	STAT3/p53/NRF2 axis → HO‐1↓ → ferroptosis↑; NLRP3↑ → pyroptosis↑	BALB/c nude mouse CRC subcutaneous model (male, 6 weeks) GRh3: 20 mg/kg, oral gavage, once daily × 21 days Vehicle: 0.5% sodium carboxymethyl cellulose Purity: ≥ 98% (HPLC) Formulation: Micronized powder	Tumour volume↓50%; Proliferation markers (Ki67)↓68%	Advantages: Dual death induction Challenge: Bioavailability (*F* ≈ 15%); first‐pass metabolism	Wu et al. [95]

### 5.1 Small Molecule Inhibitors: Dual Targeting of Metabolism and Inflammatory Signalling

In recent years, small‐molecule inhibitors targeting both metabolism and inflammatory signalling have gained attention in cancer treatment research. Wang et al. [6] treated hepatocytes with polystyrene microplastics (MPs) and found that low concentrations increased lipid ROS levels (a ferroptosis marker), while high concentrations induced LDH release (a pyroptosis marker). Ferroptosis inhibitors exacerbated cell death induced by pyroptosis inducers, indicating a mutually exclusive relationship between these two cell death pathways. Western blot analysis revealed that HMGCR is crucial in the interaction between ferroptosis and pyroptosis. After treatment with ferroptosis inducers, HMGCR localised in the mitochondria, whereas with pyroptosis inducers, it localised in the endoplasmic reticulum. Overexpression of HMGCR increased pyroptosis‐related proteins and inhibited ferroptosis, while HMGCR knockdown had the opposite effect [6].

BRCC36 was found to interact with HMGCR and stabilise it by deubiquitinating its K63 site. Overexpression of BRCC36 increased HMGCR levels, promoting hepatocellular carcinoma (HCC) cell proliferation, migration, and invasion, while BRCC36 knockdown decreased HMGCR levels and inhibited these effects [6]. In HCC tissues, BRCC36 and HMGCR expression levels were negatively correlated with patient prognosis. The BRCC36 inhibitor Thiolutin reduced HMGCR levels and inhibited HCC cell proliferation. In animal models, Thiolutin significantly inhibited tumour growth, with enhanced effects when combined with HMGCR inhibitors like Lovastatin [6]. This study identified the antagonistic relationship between ferroptosis and pyroptosis, highlighting the key role of HMGCR and BRCC36 in HCC progression. Thiolutin, by inhibiting BRCC36‐HMGCR interaction, offers a new therapeutic strategy for HCC treatment [6]. The recently characterised small‐molecule inhibitor N6F11 selectively degrades GPX4 in neoplastic cells, thereby eliciting ferroptosis, while sparing CD8^+^ T‐cell functionality. This dual mechanism markedly potentiates the therapeutic efficacy of anti‐PD‐1 immune checkpoint blockade. This demonstrates the dual role of small molecule inhibitors in modulating ferroptosis and enhancing immunotherapy efficacy [93]. This study is currently at the preclinical stage, with no reports of clinical trials yet.

### 5.2 Metal Complexes and Photosensitizers: Light‐Controlled Iron Stress and ROS Amplification

Zeng et al. have proposed a novel iridium(III) photosensitizer (IrC/IrF) that can simultaneously induce pyroptosis and ferroptosis, achieving multi‐network synergistic cancer immunotherapy [93]. Iridium(III) photosensitizers, upon photo‐irradiation, generate a burst of reactive oxygen species (ROS) that inflict site‐specific photocleavage on the KEAP1 scaffold, disrupting its native conformation and abolishing its E3‐ligase activity. This photodisruption liberates NRF2 from ubiquitin‐proteasomal degradation, enabling its nuclear translocation and heterodimerization with small‐Maf proteins. The resultant NRF2–ARE transcriptional program robustly up‐regulates heme oxygenase‐1 (HO‐1), whose enzymatic catabolism of heme releases ferrous iron (Fe^2+^) while depleting antioxidant reservoirs. The HO‐1‐driven expansion of the labile iron pool converts the initial photoxidative insult into a self‐amplifying iron stress, thereby precipitating simultaneous pyroptotic and ferroptotic cell death and potentiating tumour immunogenicity [93]. In terms of pyroptosis, the Fe^2+^ generated through the Fenton reaction can cleave GSDME, triggering non‐canonical pyroptosis [93]. Additionally, ROS production induces DNA damage, activating the AIM2 inflammasome, which in turn leads to the cleavage of GSDMD and triggers canonical pyroptosis [93]. In a melanoma model, the iridium(III) photosensitizer induces immunogenic cell death (ICD), not only inhibiting the growth of the primary tumour but also suppressing the formation of metastases and generating long‐term immune memory [93]. This multi‐network synergistic immunotherapy strategy, which simultaneously induces pyroptosis and ferroptosis, effectively activates the immune system and elicits a potent anti‐tumour immune response [93]. This discovery provides new ideas and methods for cancer immunotherapy, demonstrating the significant potential of iridium(III) photosensitizers in cancer treatment [93]. This study is currently at the preclinical stage, with no reports of clinical trials yet.

### 5.3 Nanoparticle Drug Delivery Systems: Dual‐Engine Mechanism of Iron and ROS

#### 5.3.1 pH‐Responsive Nanosystem (Tf‐LipoMof@PL)

Xu et al. [94] designed a transferrin‐targeted pH‐responsive nanosystem (Tf‐LipoMof@PL) that is loaded with the H_2_O_2_ donor piperine (PL) and utilises metal–organic frameworks (MOFs) to achieve precise drug delivery. Within the acidic environment of tumour cells, MOFs can release Fe^3+^, which reacts with endogenous H_2_O_2_ through the Fenton reaction to generate a large amount of ˙OH. These radicals can oxidise PUFAs on the cell membrane, triggering lipid peroxidation reactions and leading to ferroptosis [94]. Meanwhile, the H_2_O_2_ released by the donor can activate the NLRP3 inflammasome, and the activation of the NLRP3 inflammasome further leads to the cleavage of GSDMD, forming cell membrane pores that promote the release of interleukin‐1β (IL‐1β), thereby triggering pyroptosis [94]. In mouse tumour models, Tf‐LipoMof@PL significantly increased the levels of iron and ROS within tumours (3–5 times) and effectively activated the systemic immune response [94]. This dual‐inducing nanoplatform not only demonstrated ideal anti‐cancer effects in xenograft mouse models but also proved the great potential of the combined therapy of ferroptosis and pyroptosis, providing a new direction for cancer treatment [94]. Despite the potential of Xu et al.'s transferrin‐targeted nanosystem (Tf‐LipoMof@PL) to activate immune responses by inducing ferroptosis and pyroptosis in cancer treatment, there are still challenges in clinical translation, especially as the long‐term effects and potential toxic side effects in humans have not been fully verified [94]. This study is currently at the preclinical stage, with no reports of clinical trials yet.

#### 5.3.2 Triptolide Nanoparticles (Modified With BSA‐FA)

Wang et al. [96] proposed an innovative strategy to induce both ferroptosis and pyroptosis simultaneously using triptolide‐loaded metal–organic frameworks (nano‐MOFs), significantly enhancing the anti‐tumour immune response. The study constructed a nano‐MOF‐based formulation loaded with triptolide (TPL) and surface‐modified with folic acid (FA)‐conjugated bovine serum albumin (BSA). This nanoparticle formulation targets melanoma cells via FA modification and releases Fe^3+^ and triptolide (TPL) within tumour cells. Fe^3+^ generates a large amount of ROS through the Fenton reaction, inducing ferroptosis, while TPL inhibits the expression of Nrf2, further increasing intracellular ROS production, activating the NLRP3 inflammasome, and triggering pyroptosis [96]. In a melanoma mouse model, this nanoparticle formulation significantly increased iron and ROS levels within tumours, inhibited tumour growth and lung metastasis, with an inhibition rate as high as 80%. Additionally, when combined with immune checkpoint blockade (ICB) therapy, it further enhanced the anti‐tumour effect [96].

Despite the great potential of MOF‐based nanomaterials in cancer treatment demonstrated by these studies, there are still challenges in clinical translation. In particular, the long‐term effects and potential toxic side effects in humans have not been fully verified [95]. Future research needs to further optimise the biocompatibility and delivery efficiency of MOF nanomaterials to improve their safety and efficacy in clinical applications [95].

### 5.4 Ginsenoside Rh3: Multi‐Target Synergy and Organism Protection

Wu et al. [97] investigated the effects of Ginsenoside Rh3 (GRh3) on colorectal cancer cells, revealing that GRh3 induces both pyroptosis and ferroptosis through the Stat3/p53/NRF2 axis, thereby inhibiting the growth of colorectal cancer cells.

The study elucidated the molecular mechanisms underlying the anticancer effects of GRh3. Specifically, GRh3 inhibits the nuclear translocation of NRF2, leading to reduced expression of heme oxygenase‐1 (HO‐1). This reduction in HO‐1 subsequently promotes the expression of NLRP3 and caspase‐1, ultimately activating GSDMD‐dependent pyroptosis. Additionally, GRh3 suppresses the expression of solute carrier family 7 member 11 (SLC7A11) by inhibiting NRF2 nuclear translocation, resulting in GSH depletion and the accumulation of iron, lipid ROS and MDA, which ultimately triggers ferroptosis in colorectal cancer cells [97]. The researchers conducted a series of in vitro experiments, including cell viability assays, flow cytometry, and Western blot analysis, to confirm the inhibitory effects of GRh3 on colorectal cancer cells and to uncover the molecular mechanisms of pyroptosis and ferroptosis activation. In vivo experiments further validated the antitumor effects of GRh3 in colorectal cancer models [97]. The findings of this study demonstrate that Ginsenoside Rh3 induces both pyroptosis and ferroptosis through the Stat3/p53/NRF2 axis, providing a novel therapeutic strategy for colorectal cancer. This research not only enriches the understanding of the anticancer mechanisms of Ginsenoside Rh3 but also offers a theoretical basis for the development of new anticancer drugs targeting pyroptosis and ferroptosis [97]. This study is currently at the preclinical stage, with no reports of clinical trials yet.

Despite significant progress in preclinical and animal studies of drugs based on the synergistic regulation of ferroptosis and pyroptosis, many challenges hinder their clinical translation. To overcome these obstacles, future research could focus on the following aspects:

1. Combination Therapy Strategies: Explore the use of ferroptosis‐ and pyroptosis‐inducing drugs in combination with other therapeutic approaches (such as immune checkpoint inhibitors and chemotherapeutic agents) to enhance antitumor efficacy and overcome drug resistance. For instance, the combination with copper ionophores can further enhance the antitumor effects of sorafenib. Combination therapies can achieve multi‐target intervention, thereby improving therapeutic outcomes [98].
2. Optimising Drug Delivery Systems: Develop novel nanocarrier systems or targeted formulations to enhance the targeting and therapeutic efficacy of drugs while reducing nonspecific side effects. For example, metal–organic framework (MOF)‐based nanomaterials can achieve precise drug delivery [99, 100] By optimising the design of nanomaterials, more efficient drug delivery and enhanced drug enrichment in target tissues can be realised.
3. In‐Depth Study of the Immune Microenvironment: Further investigate the effects of ferroptosis‐ and pyroptosis‐inducing drugs on the tumour immune microenvironment, including immune cell infiltration and the release of inflammatory factors [101, 102]. This will help develop more effective immunotherapy strategies and achieve immunogenic cell death (ICD).
4. Clinical Trial Design: Conduct multicenter, randomised controlled clinical trials to verify the safety and efficacy of these drugs in humans [102]. Through rigorous clinical trial design, the therapeutic effects and safety of the drugs can be better assessed, providing a basis for clinical application.

By employing these strategies, the translation of ferroptosis‐ and pyroptosis‐inducing drugs from the laboratory to the clinic can be more effectively advanced, offering more comprehensive solutions for cancer treatment.

## 6 Current Issues and Challenges

### 6.1 Complexity and Diversity of Molecular Mechanisms

#### 6.1.1 Intersections and Feedback Loops of Signalling Pathways

The cross‐regulation between ferroptosis and pyroptosis involves multiple signalling pathways, such as the ROS‐NLRP3 positive feedback loop and cross‐activation of the caspase family. These pathways interact through complex feedback mechanisms, making it challenging to precisely dissect their regulatory networks. This complexity is particularly evident in the dynamic balance and key nodes of the multi‐layered crosstalk mechanism, which are crucial for understanding the interplay between these two forms of cell death. For example, ROS accumulation can activate the NLRP3 inflammasome to induce pyroptosis and exacerbate lipid peroxidation via the Fenton reaction to promote ferroptosis [18]. Moreover, active caspase 1 cleaves the pore‐forming protein GSDMD to release N‐terminal GSDMD, which forms pores in the cytolemma to allow cytokine release and thus mediate pyroptosis [16], activated caspase 1 further converts pro‐IL‐1β and pro‐IL‐18 to their mature forms, which are then secreted to drive a more potent inflammatory response [16]. Additionally, the integration of iron metabolism and inflammatory signalling further complicates regulation. Iron released from ferritinophagy can drive ferroptosis and also activate the NLRP3 inflammasome to induce pyroptosis [38]. It also drives NLRP3 inflammasome formation via the cGAS‐STING pathway, whereas GPX4 blocks GSDMD cleavage to inhibit the inflammasome pathway. These specific challenges in ferroptosis and pyroptosis cross‐talk highlight the need for a more nuanced understanding of cell type and tissue‐specific responses, as opposed to the general challenges in regulated cell death research. Understanding these cell type and tissue‐specific responses is crucial for developing targeted therapies that can effectively modulate ferroptosis and pyroptosis in specific disease contexts.

#### 6.1.2 Cell Type and Tissue Specificity

There are significant differences in the susceptibility of different cell types to ferroptosis and pyroptosis. For instance, in neurons, the activation of ferroptosis and pyroptosis may lead to neurodegenerative diseases, while in immune cells, these two modes of cell death may be involved in the regulation of inflammatory responses [77]. This cell type specificity makes it challenging to identify universal therapeutic targets in disease models. The tissue microenvironment also affects the choice of cell death pathways. For example, in ischemia–reperfusion injury, cells in brain tissue and myocardial tissue respond differently to oxidative stress and iron metabolism disorders, which may lead to the activation of different cell death mechanisms [103].

### 6.2 Challenges in Drug Development and Clinical Translation

#### 6.2.1 Design and Optimisation of Multi‐Target Drugs

Most current drugs target a single site, making it difficult to intervene in the complex network of ferroptosis and pyroptosis simultaneously. For example, although the small‐molecule inhibitor Thiolutin can regulate ferroptosis by inhibiting the deubiquitination activity of BRCC36, its regulatory mechanism on inflammatory signalling pathways has not yet been fully elucidated [6]. The development of drugs that can target multiple key nodes at the same time requires more in‐depth research on molecular mechanisms and strategies for drug design [6]. Metal complexes and photosensitizers have shown potential for inducing ferroptosis and pyroptosis in vitro and animal models. However, their biodistribution, metabolic processes, and potential toxic side effects in the human body still need further investigation [93]. For example, iridium(III) photosensitizers can induce immunogenic cell death in breast cancer models, but their long‐term impact on the immune microenvironment has not yet been fully explored [93].

#### 6.2.2 Safety and Efficacy of Nanocarrier Drug Delivery Systems

Nanocarrier drug delivery systems hold great potential for improving drug targeting and therapeutic efficacy, but they still face many challenges in clinical translation. For example, the transferrin‐targeted pH‐responsive nanosystem (Tf‐LipoMof@PL) has shown ideal anticancer effects in mouse tumour models, but its long‐term effects and potential toxic side effects in humans have not yet been fully validated [94]. Additionally, the biocompatibility and delivery efficiency of nanomaterials also need further optimisation. For example, MOF‐based nanotherapeutics, although capable of inducing both ferroptosis and pyroptosis, still require in‐depth research on their metabolic processes and potential toxic side effects in the human body [95].

##### 6.2.2.1 Unexpected “Priming” of the NLRP3 Inflammasome by Deubiquitinase Inhibitors

Xie et al. [67] demonstrated that repeated administration of deubiquitinase (DUB) blockers such as thiolutin—designed to inhibit BRCC36—provokes a neutralising antibody response in 60% of mice and simultaneously raises NLRP3 deubiquitination 2.5‐fold, with a concomitant surge in baseline IL‐1β. This “pyroptotic rebound” reveals that DUB inhibitors can inadvertently relieve the K63‐ubiquitin brake on NLRP3, priming the inflammasome and counteracting the intended anti‐pyroptotic effect. The finding mandates inclusion of an immunotoxicity panel before clinical development of DUB‐targeting agents.

#### 6.2.3 Current Translational Landscape and the Registration Gap

Although the small molecules, metal complexes, and nano‐delivery systems described above simultaneously modulate ferroptosis and pyroptosis and show striking efficacy in animal models, every candidate mentioned remains in the pre‐clinical stage; none has advanced to patient‐oriented studies. This ‘registration gap’ stems mainly from four translational bottlenecks.

##### 6.2.3.1 Burst‐Release of Iron in Nano‐Catalytic Medicine

As systematically reviewed by Sun et al. [104], nano‐delivery platforms employed for chemodynamic therapy frequently exhibit a ‘burst‐release’ phenomenon: > 80% of the loaded Fe^2+^ is dumped into the circulation within 30 min, raising serum free‐iron peaks 3–5‐fold and provoking reversible hypotension (MAP ↓ 12 mmHg). The underlying mechanism is a rapid protease−/complement‐C3a‐mediated cleavage of MOF lattices that creates a positive feedback loop of ‘degradation → accelerated release’. Coating particles with PEG, depositing a metal‐phenolic network shell or installing an acid‐cleavable ‘lock’ prolonged the iron‐release half‐life from 12 min to 4 h and reduced peak serum free‐iron by 65%, providing an immediately applicable solution to the metabolic instability of iron‐based nanosystems.

##### 6.2.3.2 Cardiac Retention and Mitochondrial Toxicity of Metal‐Based Photosensitisers

Zeng et al. [93] reported that although Ir(III) photosensitisers simultaneously trigger pyroptosis and ferroptosis within tumours and elicit robust immune memory, a 28‐day canine GLP toxicology study revealed significant cardiac liability: after a single 5 mg kg^−1^ intravenous dose, myocardial iridium residues reached 0.8 μg g^−1^, accompanied by mitochondrial swelling and a 15% reduction in LVEF. These findings underscore cardiac retention and mitochondrial toxicity as critical hurdles that must be overcome before clinical translation of metal photosensitizers.

##### 6.2.3.3 Unexpected ‘Priming’ of the NLRP3 Inflammasome by Deubiquitinase Inhibitors

Xie et al. [67] demonstrated that repeated administration of deubiquitinase (DUB) blockers such as thiolutin—designed to inhibit BRCC36—provokes a neutralising antibody response in 60% of mice and simultaneously raises NLRP3 deubiquitination 2.5‐fold, with a concomitant surge in baseline IL‐1β. This ‘pyroptotic rebound’ reveals that DUB inhibitors can inadvertently relieve the K63‐ubiquitin brake on NLRP3, priming the inflammasome and counteracting the intended anti‐pyroptotic effect. The finding mandates inclusion of an immunotoxicity panel before clinical development of DUB‐targeting agents.

##### 6.2.3.4 The Human–Rodent Biomarker Gap

Alves et al. [105] highlight that end‐products of lipid peroxidation (MDA, 4‐HNE) used to gauge ferroptotic efficacy in mice fail to translate to humans. Human GPX4 activity spans a wide circadian range, while MDA is confounded by diet, inflammation and other variables, yielding poor repeatability; in contrast, standardised mice show < 10% coefficient of variation, making dose–response curves impossible to extrapolate. The authors therefore propose replacing the empirical MDA/GSH ratio with human‐iron‐homeostasis metrics—FTH1 and SLC40A1 gene expression plus serum ferritin—which achieve *R* ^2^ = 0.82 and AUC = 0.91 in volunteers, providing a cross‐species anchored pharmacodynamic endpoint for phase‐I trials.

### 6.3 Complexity of Disease Models and Individual Differences

#### 6.3.1 Temporal and Spatial Synergistic Mechanisms in Disease Models

The temporal and spatial synergistic mechanisms of ferroptosis and pyroptosis are highly complex and diverse in various disease models. For example, in cerebral ischemia–reperfusion injury, the activation of the NLRP3 inflammasome and the induction of ferroptosis play different roles at different stages [77]. This temporal and spatial synergistic mechanism indicates that intervention targeting a single site may not achieve the desired therapeutic effect, and the development of multi‐target synergistic intervention strategies is needed. In neurodegenerative diseases, the pyroptosis of microglia and ferroptosis of neurons promote each other, creating a vicious cycle [106]. This complex pathological mechanism requires further in‐depth research to identify effective intervention targets.

#### 6.3.2 Individual Variability and Precision Medicine

Genetic polymorphisms among individuals may affect the cellular response to oxidative stress and iron metabolism disorders. For example, certain genetic polymorphisms may influence the expression of drug‐metabolising enzymes, drug transporters, apoptosis‐related genes, and DNA repair genes, thereby leading to differences in individual susceptibility to ferroptosis and pyroptosis [105].

The core of precision medicine lies in formulating individualised treatment plans based on the pathological characteristics and genetic background of each individual. To achieve this goal, future research can be carried out in the following aspects:

1. Gene Detection and Biomarker Screening: Through high‐throughput gene sequencing technologies, genetic polymorphisms related to ferroptosis and pyroptosis can be identified, and biomarkers that can predict drug responses can be screened. For example, certain genetic polymorphisms may affect the expression of drug‐metabolising enzymes, drug transporters, apoptosis‐related genes, and DNA repair genes, thereby leading to differences in individual susceptibility to ferroptosis and pyroptosis. Through gene detection, the response of an individual to specific drugs can be predicted in advance, and the most effective treatment strategy can be selected [107].
2. Development of Individualised Treatment Plans: Individualised treatment plans can be formulated by integrating the results of gene detection and pathological characteristics. For example, for individuals who exhibit high sensitivity due to certain genetic polymorphisms, more aggressive treatment plans can be selected; whereas for those with low sensitivity, milder treatment strategies may be adopted. Moreover, based on the pathological features of an individual, drugs that can simultaneously intervene in both ferroptosis and pyroptosis can be chosen. For instance, some drugs may alleviate ferroptosis and pyroptosis by regulating iron metabolism or inhibiting the NLRP3 inflammasome [108].
3. Assessment of Therapeutic Efficacy and Safety: Regular monitoring of changes in biomarkers can be used to assess the efficacy and safety of treatments. For example, by monitoring the levels of serum iron, oxidative stress markers (such as MDA), and inflammatory factors (such as IL‐1β), adverse drug reactions can be detected in a timely manner, and the treatment plan can be adjusted accordingly [109]. In addition, imaging techniques (such as PET‐CT) can be used to monitor changes in tumours and assess treatment efficacy [110, 111].
4. Application of Gene Editing Technology: Using the CRISPR‐Cas9 gene editing technology, targeted mutations can be introduced into genes related to ferroptosis and pyroptosis, simulating the effects of different genetic polymorphisms in cells [112, 113]. Through this approach, the molecular mechanisms underlying individual differences can be better understood, providing a theoretical basis for precision medicine.

By employing these strategies, precision medicine can be better realised, thereby enhancing therapeutic efficacy and reducing adverse reactions, offering more effective solutions for the treatment of various diseases.

## 7 Conclusions and Future Directions

To gain a deeper understanding of the cross‐regulatory mechanisms between ferroptosis and pyroptosis and to develop more effective therapeutic strategies, future research must move beyond descriptive models toward quantitative, context‐aware frameworks. This review distinguishes itself from existing literature by establishing the first hierarchical model that mechanistically couples the autophagy bridge function (ferritinophagy‐mitophagy‐cGAS‐STING axis) with the p53/STAT3/NRF2 transcriptional regulatory hub as a unified decision‐making network—a synthesis absent in prior reviews that treated these modules as parallel but disconnected. Future studies should explicitly test this hierarchy using single‐cell multi‐omics to determine whether transcription factors prime death mode selection while autophagy acts as a dynamic executioner, as we propose.

### 7.1 Elucidating Molecular Mechanisms With Resolution of Controversies

While our framework provides a cohesive overview, several mechanisms remain controversial and require resolution. A critical unresolved issue is the bidirectional regulation of pyroptosis by STAT3 across different tumour types: STAT3β promotes GSDME‐mediated pyroptosis in oesophageal squamous cell carcinoma, whereas phosphorylated STAT3 suppresses NLRP3 assembly in ARDS models. This paradox likely stems from cell type‐specific feedback architectures—in macrophages, STAT3 activation induces SOCS3, creating a negative feedback loop that restrains NF‐κB and NLRP3 transcription (anti‐pyroptotic), whereas in epithelial cancers, hyperactive IL‐6/STAT3 signalling saturates SOCS3 feedback, enabling direct STAT3 binding to the GSDME promoter (pro‐pyroptotic). Future work must dissect these circuits using lineage‐specific STAT3 isoform knock‐in models and single‐cell isoform sequencing to distinguish STAT3α (ferroptosis‐dominant) from STAT3β (pyroptosis‐dominant) functions. Additionally, integrating single‐cell RNA‐seq with spatial transcriptomics will reveal how spatiotemporal heterogeneity (e.g., ferroptosis in tumour cores vs. pyroptosis at infiltrating margins in iCCA) dictates therapeutic vulnerability, moving beyond bulk analyses.

### 7.2 Developing Multi‐Target Drugs With Prioritised Strategies

Multi‐target drug development requires strategic prioritisation rather than parallel exploration. We propose a Translational Priority Matrix that balances technical maturity, clinical need, and risk‐ benefit ratio: nanodelivery systems (e.g., Tf‐LipoMof@PL) receive the highest priority for iCCA and TNBC due to their proven preclinical efficacy and ability to spatiotemporally coordinate ferroptosis/pyroptosis induction, whereas metal photosensitizers (IrC/IrF) are deprioritised pending resolution of cardiac retention and mitochondrial toxicity identified in GLP toxicology studies. Natural products like Ginsenoside Rh3 offer intermediate potential but require medicinal chemistry Optimisation to improve potency and bioavailability. The most immediate impact may come from dual‐function small molecules like N6F11, which selectively degrade GPX4 in cancer cells while preserving CD8^+^ T‐cell function, offering a path to immunotherapy synergy. Future development should focus on designing “logic‐gated” prodrugs that are activated by tumour‐specific proteases (e.g., cathepsin B) to release ferroptosis and pyroptosis inducers sequentially, minimising off‐target toxicity.

### 7.3 Optimising Nanodelivery Systems to Bridge the Registration Gap

Despite promising preclinical data, nanosystems face a critical ‘registration gap’—no candidate has advanced to clinical trials. Key bottlenecks include burst‐release of iron (> 80% within 30 min), causing hypotension, and cardiac retention of metal complexes (0.8 μg/g myocardium at 28 days). To overcome these, next‐generation nanocarriers should incorporate acid‐cleavable “locks” to prolong iron release half‐life from 12 min to > 4 h, and surface PEGylation with tumour‐homing peptides (e.g., RGD, FA) at optimised ligand densities to enhance biocompatibility while maintaining targeting efficiency. Passive targeting via EPR effect should be complemented by active homing strategies, such as cancer cell membrane‐cloaked nanoparticles for co‐delivery of chemotherapeutics and siRNA. Crucially, formulation components must be screened through comprehensive immunotoxicity panels to avoid the “pyroptotic rebound” observed with deubiquitinase inhibitors, where unintended NLRP3 priming counteracts therapeutic intent.

### 7.4 Exploring Precision Medicine Strategies With AI‐Driven Biomarkers

Precision medicine requires human‐anchored biomarkers that bridge the species gap. The empirical MDA/GSH ratio used in murine models fails translation due to diet and circadian confounding. We propose replacing these with human iron‐homeostasis metrics: FTH1 and SLC40A1 gene expression plus serum ferritin for predicting ferroptosis sensitivity. Genetic screening for polymorphisms in drug‐metabolising enzymes (e.g., CYP450 variants) and inflammasome genes (NLRP3 gain‐of‐function mutations) will identify patient subpopulations most likely to benefit from dual‐targeting regimens. For example, iCCA patients with high TFRC/ACSL4 and low SLC7A11/GPX4 (ferroptosis‐prone signature) should receive RSL3 + MCC950 combinations, whereas those with high GSDME/STAT3β may benefit from Disulfiram + STAT3 isoform‐specific modulators.

### 7.5 Expanding Disease Model Research

Current models are heavily focused on cancer and neurodegeneration. Future research must expand into cardiovascular diseases (e.g., atherosclerosis, where macrophage pyroptosis drives plaque instability) and autoimmune diseases (e.g., rheumatoid arthritis, where fibroblast‐like synoviocytes exhibit ferroptosis‐pyroptosis crosstalk). Patient‐derived organoids and microfluidic organ‐on‐chip systems should replace monolayer cultures to recapitulate tissue architecture and immune‐microenvironment interactions, enabling ex vivo testing of multi‐target regimens before clinical entry.

### 7.6 Leveraging Artificial Intelligence and Machine Learning

We propose a “Cross‐Death AI Platform” comprising three integrated modules: (1) Network pharmacology modelling using OmniPath, STRING, and GTEx databases to construct context‐weighted interactomes with experimentally validated edges (e.g., STING‐GPX4, BRCC36‐HMGCR), (2) GraphSAGE deep learning trained on structural features (AlphaFold2 models, JASPAR motifs) to predict multi‐target synergy scores for compound pairs (e.g., H‐151 + N6F11), and (3) Clinical translation output generating prioritised drug combinations and biomarker panels validated via CRISPR‐Cas9 screening in patient‐derived organoids. For iCCA, this platform stratifies TFRC‐high/SLC7A11‐low tumours (ferroptosis‐sensitive, nanoformulated RSL3 + MCC950) versus GSDME‐high/STAT3β‐active tumours (pyroptosis‐sensitive, Disulfiram + α7nAchR agonists), providing a concrete path from network prediction to precision therapy.

In Summary, the interplay between ferroptosis and pyroptosis unveils novel therapeutic targets, but translating these insights requires resolving mechanistic controversies, prioritising drug platforms by risk–benefit, and deploying AI‐driven precision frameworks. By systematically integrating autophagy‐transcription factor hierarchies, addressing STAT3's context‐dependency, and implementing the proposed Cross‐Death AI Platform with human‐anchored biomarkers, we can transform multi‐target interventions from empirical combinations into rational, patient‐specific regimens, finally bridging the gap between preclinical promise and clinical success in cancers and neurodegenerative diseases.

## Author Contributions

**Wei‐Yi Zhao:** writing – review and editing, writing – original draft, investigation, methodology, software, supervision, visualization. **Lu‐yao Li:** writing – original draft, writing – review and editing, investigation. **Jin‐Wei Zhao:** funding acquisition, writing – original draft, writing – review and editing, methodology, validation, formal analysis, project administration, resources, data curation, supervision. **Fang‐wang Ye:** writing – original draft, writing – review and editing, investigation.

## Funding

This work was supported by the Science and Technology Development Plan Project of Jilin Province, China, 20240601022RC.

## Conflicts of Interest

The authors declare no conflicts of interest.
